# Bis-Pyrene-Appended Polyvinyl Chloride: Mechanosynthesis and Nitro-Explosive Detection

**DOI:** 10.3390/s26175342

**Published:** 2026-08-24

**Authors:** Vadim A. Platonov, Leila K. Sadieva, Mikhail A. Makovskiy, Nikolay A. Belyaev, Tatiana I. Shendrikova, Artem V. Baklykov, Dmitry S. Kopchuk, Igor S. Kovalev, Grigory V. Zyryanov, Valeriy N. Charushin

**Affiliations:** 1 Institute of Chemical Engineering, Ural Federal University, 19, Mira Str., Yekaterinburg 620002, Russia; vplatonov10@mail.ru (V.A.P.); ma-makovsy@mail.ru (M.A.M.); n.a.beliaev@urfu.ru (N.A.B.); t.i.shenderikova@urfu.ru (T.I.S.); art.baklykov@gmail.com (A.V.B.); dkopchuk@mail.ru (D.S.K.); ekls85@yandex.ru (I.S.K.); g.v.zyrianov@urfu.ru (G.V.Z.);; 2 I. Ya. Postovsky Institute of Organic Synthesis of RAS (Ural Division), 22/20, S. Kovalevskoy/Akademicheskaya Str., Yekaterinburg 620137, Russia

**Keywords:** bis-pyrene, PVC, mechanosynthesis, ball-milling, green chemistry, chemosensors, fluorescence “turn-off” response, nitro compounds, explosive detection

## Abstract

The remote detection of nitro compounds typically requires complex analytical procedures and expensive instrumentation, often resulting in prolonged analysis times. Fluorescence-based methods, which exploit the quenching of organic fluorophores in the presence of nitro analytes, offer a more practical and efficient alternative. Particularly promising is the use of excimer emission generated by bis-pyrene species (bis-Pyr), formed either through supramolecular interactions or covalent linkages. Regarding the latter, the integration of covalently linked bis-Pyr sensors onto polymer backbones, such as commercially available polyvinyl chloride (PVC), represents a highly attractive strategy. In this manuscript, we wish to report our recent findings on the mechanosynthesis of bis-pyrene-based PVC-supported chemosensors, and on studies of its fluorescence response towards common nitro-compound/explosive components, such as 2,4-dinitrotoluene (DNT), 2,4,6-trinitrotoluene (TNT), taggant 2,3-dimethyl-2,3-dinitrobutane (DMDNB), pentaerythritol tetranitrate (PETN) and 2,4,6-trinitrophenol (picric acid, PA). For DNT, a selective fluorescence “*turn-off*” response was observed with a Stern–Volmer quenching constant as high as 1.15 × 10^4^ M^−1^.

## 1. Introduction

The high demand of materials and methods for the remote, rapid and highly selective detection of nitro-containing explosives and their blends, as well as the contamination of water resources (reservoirs, rivers, and groundwater) and soils with nitro compounds—specifically explosives and their degradation products (in conflict zones and areas near defense industry facilities) [1], as well as herbicides and pharmaceuticals [2]—are pressing challenges for the sustainable development of modern society.

The detection of nitro compounds often relies on complex, multi-step approaches [3] and expensive instrumentation, leading to long analysis times [4]. These limitations hinder the widespread application of such methods. Fluorescence-based methods are among the most promising alternatives for detecting analytes. They offer significant advantages, including low cost, simplicity of use, high selectivity, and fast response times, utilizing fluorescent chemosensors based on small molecules or polymeric materials derived from them.

Among these materials, structures based on polycyclic aromatic hydrocarbons (PAHs)—especially pyrene—are particularly noteworthy due to their unique photophysical properties: high quantum yield, long fluorescence lifetimes [5], and high sensitivity of their emission to the changes in the local environment [6,7,8], which, in combination with their electron-donor properties, makes them very responsive to the presence of electron-deficient analytes, such as nitro-containing compounds (key components of explosive devices) [9].

Under conditions that promote π–π stacking of PAH moieties, particularly in the case of pyrene, besides the existence of the typical monomeric emission appearance of a red-shifted, broad-band, structureless one is also observed. Most often such conditions include: (1) a high concentration of PAH units; (2) an “unfriendly” polar environment, which promotes migration of non-polar PAH units close to each other due to hydrophobic attraction; and (3) attaching of PAH units to templates (e.g., linkers, calixarenes, polymer backbones) or the entrapment of PAH units within a matrix (e.g., gel, film), which would ensure spatial proximity and optimal overlap.

This broad-band emission could be attributed either to the formation of excimers or pre-associated dimers [10]. The latter is accompanied by aggregation and is usually considered undesirable, as it leads to the decrease in fluorescence efficiency called “aggregation-caused quenching” (ACQ). The former, on the other hand, is beneficial, as excimer-based emissions are very sensitive to spatial arrangement and changes in the microenvironment and can be easily disrupted, which can be effectively exploited for analytical purposes—for instance, for the “*turn-off*” detection (*via* fluorescence quenching) of nitro compounds [11,12]. Aggregates formation can be avoided by means of the creation of steric hindrance *via* introduction of bulky groups into molecular structure of PAHs or by ensuring optimal stacking *via* molecular geometry optimization [13]. An alternative approach is the targeted design of sensory materials, which are able to exhibit the opposite phenomenon—aggregation-induced emission (AIE) [14].

Keeping in mind all mentioned above, we decided to create polymer-basedchemosensor bearing bola-type pyrenyl pendants [15] and study its ability for the detection of nitro-containing analytes *via* excimer emission quenching. Polyvinyl chloride (PVC) was chosen as a backbone for this polymer-supported chemosensor, as from one side it is widely available and cheap material and, from another side an uncontrolled emission of PVC/PVC-based materials into environment requires a developing of methods for their utilization/recycling/upcycling [16,17,18,19,20], for instance, by means of post-modification [21].

To the best of our knowledge, no reports were published so far on obtaining PVC-based bis-pyrene-containing chemosensors for the detection of explosives. The only example of PVC-based chemosensor for the fluorescence-based detection of explosives to date is a lawsone-modified PVC reported by us previously [22]. Other literature examples of PVC-based chemosensors for the explosives detection are colorimetric ones [23,24].

In this manuscript we wish to report our recent findings on mechanosynthesis of bis-pyrene-based PVC-supported fluorometric chemosensor under ball-milling conditions and studies of its fluorescence response towards common nitro-compound/explosive components, such as 2,4-dinitrotoluene (DNT), 2,4,6-trinitrotoluene (TNT), 2,3-dimethyl-2,3-dinitrobutane (DMDNB), pentaerythritol tetranitrate (PETN) and 2,4,6-trinitrophenol (picric acid, PA) in solution.

## 2. Materials and Methods

All reagents were purchased from commercial sources and used without further purification. ^1^H NMR spectra were recorded on a Bruker Avance-400 spectrometer (Bruker, Bremen, Germany), at 298 K, with the digital resolution of ±0.01 ppm. ^13^C NMR spectra were acquired at 25 °C from solutions in DMSO-d6 on a Bruker AVANCE NEO–600 (Bruker, Bremen, Germany) (151 MHz for ^13^C MHz) NMR spectrometer equipped with CryoProbeProdigy (Bruker, Bremen, Germany), and Topspin 4.0.5 software was used for all NMR data acquisition. Chemical shifts were referenced to the residual peaks of solvent as an internal standard: 2.50 ppm for ^1^H NMR and 39.52 ppm for ^13^C NMR in DMSO-d_6_, 8.05 ppm ^1^H NMR in DMF-d_7_.

Mass spectrometric studies were performed on an Agilent 6545 Q-TOF LC/MS (Agilent Technologies, Santa Clara, CA, USA) quadrupole time-of-flight mass spectrometer with an electrospray ionization source in the positive (negative) ion mode.

Melting points are not corrected.

Ball-milling experiments were carried out on a Retsch PM 100 CM ball mill (Retsch, Han, Germany) in 25 mL stainless still milling jar loaded with 3 stainless still milling balls of 10 mm diameter. FT-IR spectra were measured using a LUMOS-Bruker IR-Fourier spectrometer (Bruker, Bremen, Germany).

GPC measurements were carried out on an Agilent 1200 chromatograph with an evaporative light scattering detector (ELSD) (Agilent technologies, Santa Clara, CA, USA). UV-Vis absorption, emission and excitation spectra were obtained and titration experiments were carried out on a Solar CM2203 spectrofluorometer (Solar, Minsk, Belarus).

The starting compound (1E,4E)-1,5-di(pyren-1-yl)penta-1,4-dien-3-one **1** was synthesized according to a previously published procedure [15].

### 2.1. Synthesis of 1,5-di(pyren-1-yl)pentan-3-one (***2***)

1,5-Bis(pyren-1-yl)penta-1,4-dien-3-one **1** (2.40 g, 4.96 mmol) was dissolved in 1,4-dioxane (50 mL), and Pd/C (10% mass content, 0.30 g) was added. The resulting suspension was placed in a sealed reactor charged with hydrogen under a pressure of 5 atm and treated at 60 °C for 2 h. After that the reaction mixture was filtered to remove the catalyst, and the filtrate was evaporated to dryness. The residue was redissolved in ethyl acetate (10 mL). The organic extract was washed with water (3 × 5 mL), dried over Na_2_SO_4_, filtered and concentrated under reduced pressure. The product was purified by flash column chromatography on silica gel (Alfa Aesar Silica Gel 60 (Thermo Fisher Scientific, Waltham, MA, USA)) using dichloromethane–hexane (1:1, *v*/*v*) as an eluent. The combinate eluates were evaporated under reduced pressure, and the residue was recrystallized from toluene. Obtained white crystals of the 1,5-di(pyren-1-yl)pentan-3-one were stored in the dark at room temperature. Yield: 1.46 g (3 mmol, 60%). m.p. = 196–198 °C. NMR ^1^H (400 MHz, CDCl_3_ δ, ppm) 2.88–2.92 (m, 4H, -CH_2_-), 3.59–3.63 (m, 4H, -CH_2_-), 7.78–7.79 (m, 2H, Pyr), 7.94–7.95 (m, 1H, Pyr), 7.96–7.98 (m, 4H, Pyr), 7.99–8.02 (m, 5H, Pyr), 8.07–8.09 (m, 2H, Pyr), 8.12–8.17 (m, 4H, Pyr). NMR ^13^C (150 MHz, DMSO-d6 δ, ppm) 26.80 (2C), 43.72 (2C), 123.21 (2C), 124.09 (2C), 124.16 (2C), 124.84 (2C), 124.92 (2C), 125.00 (2C), 126.16 (2C), 126.57 (2C), 127.33 (2C), 127.42 (2C), 128.00 (2C), 128.2 (2C), 129.35 (2C), 130.37 (2C), 130.88 (2C), 135.64 (2C), 208.94. FTIR, ν/cm^−1^: ν(=C-H-) 3042; ν(-C-H) 2930; ν(C=O) 1718; ν(C=C) 1600, 1584, 1508. HRMS-ESI m/z (%): Calcd. for C_37_H_26_O [M+Na]^+^ = 509,1876; found 509.1872 (100%).

### 2.2. Synthesis of 1,5-di(pyren-1-yl)pentan-3-one oxime (***3***)

Hydroxylamine hydrochloride (0.07 g, 1.0 mmol) and sodium acetate (0.06 mg, 0.74 mmol) were dissolved in distilled water (1.5 mL). Resulting solution was added dropwise upon stirring to a suspension of 1,5-di(pyren-1-yl)pentan-3-one (0.18 g, 0.37 mmol) in DMF (10 mL) at 55 °C. The mixture was stirred at the same temperature for an additional 1 h until a complete dissolution. The resulted solution was cooled to room temperature and then poured with stirring into distilled water (50 mL). The precipitate formed was filtered off and washed with water (3 × 5 mL). The crude product was purified by flash column chromatography on silica gel (Alfa Aesar Silica Gel 60 (Thermo Fisher Scientific, Waltham, MA, USA)) using dichloromethane as the eluent. The combined eluates were evaporated under reduced pressure, yielding 1,5-di(pyren-1-yl)pentan-3-one oxime as colorless crystals.

Yield: 0.14 g (0.27 mmol, 74%). m.p. 193–195 °C. NMR ^1^H (400 MHz, DMSO-d_6_, δ, ppm): 2.41–2.44 (m, 2H, -CH_2_-), 2.81–2.83 (m, 2H, -CH_2_-), 2.81–2.83 (m, 2H, -CH_2_-), 2.37–2.40 (m, 7H, -CH_2_- +H_2_O), 3.55–3.57 (m, 2H, -CH_2_-), 7.72–7.74 (m, 1H, Pyr), 7.77–7.79 (m, 1H, Pyr), 7.84–7.86 (m, 1H, Pyr), 7.92–7.93 (m, 1H, Pyr), 8.00–8.02 (m, 1H, Pyr), 8.05–8.10 (m, 6H, Pyr), 8.12–8.13 (m, 1H, Pyr), 8.15–8.18 (m, 2H, Pyr), 8.20–8.21 (m, 1H, Pyr), 8.25–8.28 (m, 2H, Pyr), 8.40–8.41 (m, 1H, Pyr), 10.75–10.77 (s, 1H, =N-OH). NMR ^1^H (400 MHz, DMF-d_7_, δ, ppm): 2.58–2.62 (m, 2H, -CH_2_-Pyr), 2.77–2.81 (m, 7H, -CH_2_-Pyr + _n_DMF), 2.98–3.02 (m, 2H, -CH_2_-C=N-O), 3.71–3.75 (m, 2H, -CH_2_-C=N-O), 7.91–7.94 (m, 2H, Pyr), 8.02–8.10 (m, 2H, Pyr + _n_DMF), 8.11–8.15 (m, 2H, Pyr), 8.16–8.21 (m, 3H, Pyr), 8.22–8.31 (m, 4H, Pyr), 8.33–8.35 (m, 1H, Pyr), 10.95 (s, 1H, =N-OH). NMR ^13^C (150 MHz, DMSO-d_6_ δ, ppm) 28.68, 29.50, 30.26, 36.33, 122.80, 123.38, 124.02, 124.08, 124.21, 124.23, 124.85 (2C), 124.87, 124.93, 125.01 (2C), 126.09, 126.18, 126.49, 126.59, 127.09, 127.32 (2C), 127.39, 127.49 (2C), 127.84, 128.26, 129.28, 129.45, 123.24, 130.47, 130.82, 130.96, 135.91, 136.11, 157.51. FTIR, ν/cm ^−1^: ν(O-H (br.)) 3317–3112; ν(C-C) 2879; ν(C=N) 1662; ν(C=C) 1600, 1586, 1486; ν(C-H) 1455; ν(N-O) 944. HRMS-ESI m/z (%): Calcd. for C_37_H_27_NO [M+H]^+^ = 502.2165; found 502.2165 (100%).

### 2.3. Synthesis of Polymer (***4***)

A 25 mL stainless steel grinding jar containing three steel balls (d = 10 mm) was charged with the 1,5-di(pyren-1-yl)pentan-3-one oxime **3** (0.86 g, 0.17 mmol), polyvinyl chloride (0.26 g), K_2_CO_3_ (0.4 g, 2.7 mmol), and DMF (4 drops). The mixture was stirred at 500 rpm for 4 h. The resulting reaction mixture was then suspended in a methanol/water mixture (2:1, *v*/*v*, 20 mL), and the suspension was filtered, washed with water (2 × 5 mL),methanol (5 mL), and air-dried for 12 h. Subsequently, the precipitate was suspended in methanol (20 mL) containing KOH (0.18 g, 3 mmol), and the resulting mixture was heated at reflux for 3 h. The precipitate was collected by filtration, washed with water (3 × 5 mL) and methanol (3 × 5 mL), and dried at room temperature for 24 h. Polymer **4** was obtained as a light brown solid.

Yield 0.25 g (72%). m.p. = 133–135 °C. NMR ^1^H (400 MHz, DMF-d_7_, δ, ppm): 2.13–2.70 (m, 98H, -(CH_2_)_n_-(PVC) + -CH_2_-Pyr)), 2.79–2.81 (m, 77H, -CH_2_-Pyr + _n_DMF), 2.96–2.98 (m, 77H, -CH_2_-C=N-O- + _n_DMF), 3.71–3.75 (m, 2H, -CH_2_-C=N-O-), 4.44–4.70 (m, 44H, -(Cl-CH-)n-(PVC)), 7.92–7.96 (m, 2H, Pyr), 8.03–8.10 (m, 31H, Pyr + _n_DMF), 8.12–8.14 (m, 2H, Pyr), 8.18–8.20 (m, 2H, Pyr), 8.24–8.31 (m, 2H, Pyr), 8.34–8.36 (m, 2H, Pyr), 8.58–8.60 (m, 1H, Pyr). FTIR, ν/cm ^−1^: ν(C-C) 2913; ν(C=N) 1650; ν(C=C) 1558, 1540, 1508; ν(C-H) 1455; ν(N-O) 961; ν(C-Cl) 846. GPC (DMF), M_n_ = 150 kDa (PDI = 1.3).

### 2.4. Spectroscopic Studies

Organic solvents (DMF, DMSO) used for preparation of solutions were “For HPLC & Spectroscopy” grade (Alpha Chemika, Mumbai, India) and are commercially available. The water used for preparation of solutions was purified using a Millipore Simplicity UV type I high purification system (Merck, Darmstadt, Germany).

UV-Vis absorption, emission and excitation spectra were obtained and titration experiments were carried out on a Solar CM2203 spectrofluorometer (Solar, Minsk, Belarus).

The graphic processing of the data was performed using the OriginPro 2021 software (OriginLab Corporation, Northampton, MA, USA, 64-bit, version 9.8.0.200).

The fluorescence response of the compounds towards nitro-containing quenchers was quantitatively evaluated by calculation of Stern-Volmer quenching constant using the Stern-Volmer static quenching model according to Equation (1) and quenching efficiency (QE) was quantitatively estimated with the use of Equation (2):

(1) $$\frac{I_{0}}{I}=1+K_{SV}\times \left[Q\right]$$

where I_o_ is the intensity of fluorescence in the absence of a quencher, I is the intensity of fluorescence in the presence of a quencher, K_SV_ is the Stern-Volmer quenching constant for complex formation, and [Q] is the concentration of the quencher. The quenching constant (K_SV_) was calculated as the slope of the graph intensity ((I_0_/I) − 1) versus the concentration of the quencher ([Q]).

(2) $$QE(\%)=\frac{I_{0}-I}{I_{0}}\times 100$$

where I_0_ is the intensity of fluorescence in the absence of a quencher, I is the intensity of fluorescence in the presence of a quencher.

The limit of detection (LOD) was determined based on the data from fluorescence quenching experiments in accordance with the method published earlier [25]. To obtain the equation of the regression curve, a calibration graph of the sensor fluorescence intensity versus the analyte concentration was plotted. The limit of detection was calculated using the Equation (3):

(3) $$LOD=\frac{3\sigma }{k}$$

where σ is the standard deviation of the fluorophore intensity in the absence of the analyte obtained using the STEYX function in Excel (Microsoft, Redmond, WA, USA, version 2505, build 18827.20176), k is the slope of the calibration curve.

For Inner Filter Effect (IFE) correction Parker’s method improved by Lakowicz (see Equation (4)) [26] were used:

(4) $$F_{corr}=F_{obs}\times antilog\left(\frac{{OD}_{ex}+{OD}_{em}}{2}\right)$$

where F_corr_ и F_obs_ are the corrected and observed fluorescence intensities, OD_ex_ и OD_em_ are the absorption at the excitation and emission wavelengths, respectively.

## 3. Results and Discussion

### 3.1. Synthesis and Characterization

**Scheme 1:**
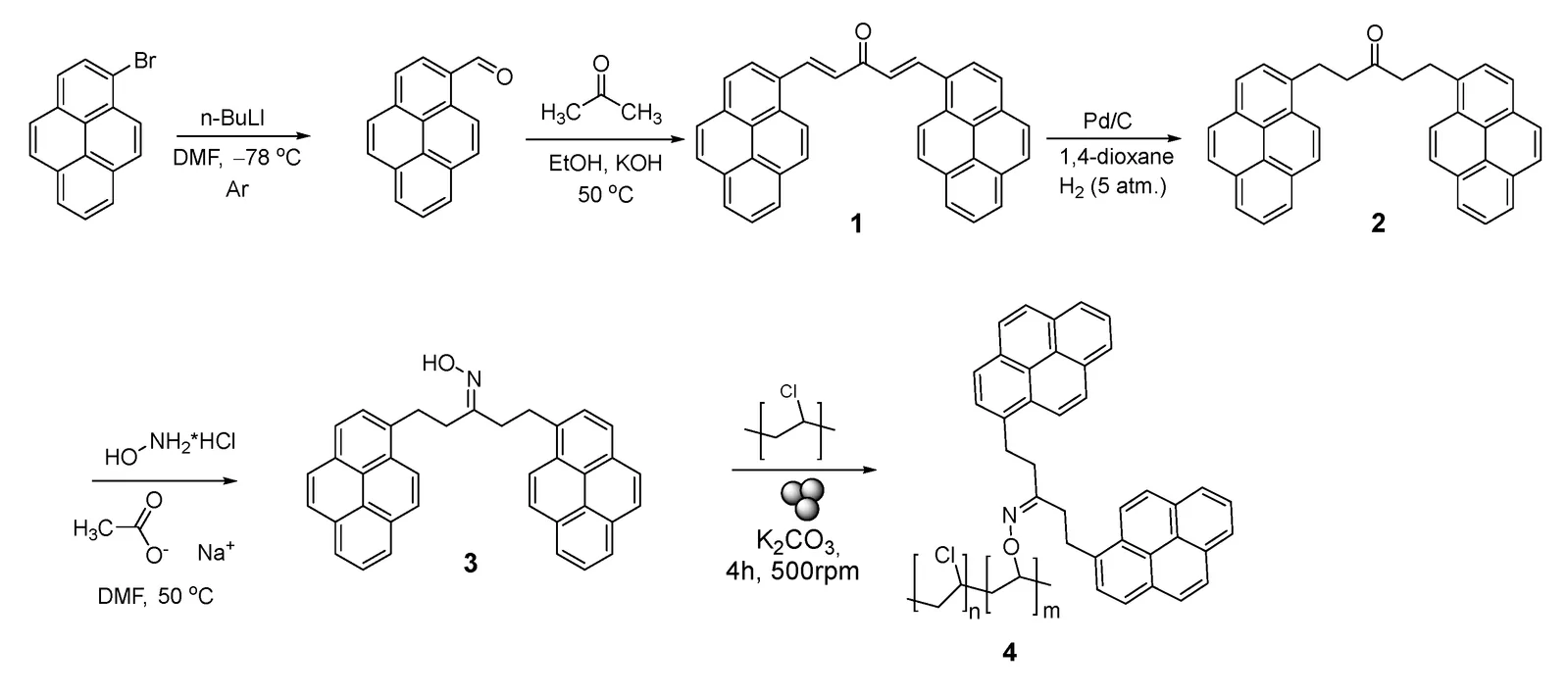
Synthesis of compounds **3**, **4**.

The starting compounds were prepared as reported earlier. Thus, by using croton condensation, we synthesized penta-1,4-dien-3-one **1** bearing pyrene moiety (Scheme 1). The following reduction of double bonds resulted in 1,5-di(pyren-1-yl)pentan-3-one **2**. And by the reaction of **2** with hydroxylamine hydrochloride in the presence of NaOAc the bispyrene-oxime **3** was obtained. Oximes are known to react readily with alkylhalides to form oxime ethers [27]. And we have assumed the possibility of involving polyvinyl chloride (PVC) in a similar reaction with bispyrene-oxime **3** under ball-milling conditions at a final step. Thus, according to our earlier reports [22] by means of reaction between oxime **3** and commercially available PVC under ball-milling conditions at 500 rpm for 4 h in the presence of potassium carbonate, which was used both as a base and as an additional abrasive, the target bispyrene-appended PVC **4** was obtained. And DMF (four drops) was used as a co-solvent to provide better homogenization of the reaction mixture according to a Liquid Assisted Grinding (LAG) technique (Scheme 1).

**Scheme 2:**
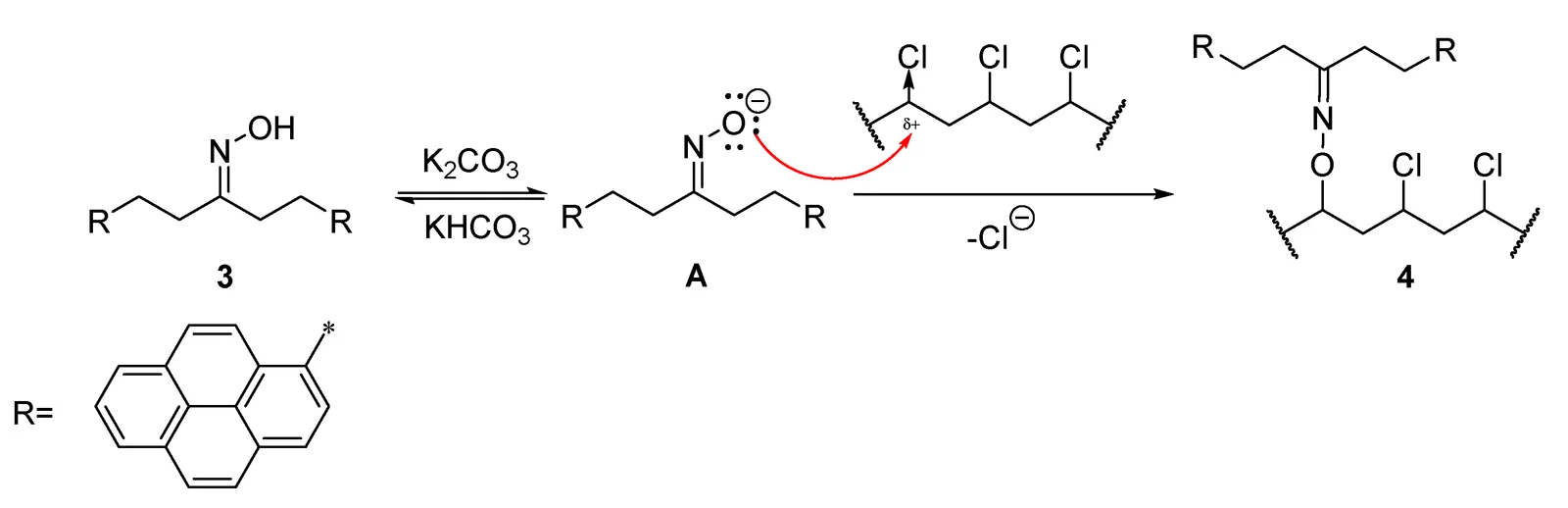
Proposed mechanism of the PVC reaction with compound **3**.

The mechanism of the reaction above is a typical nucleophilic *ipso*-substitution reaction. The first stage involves the formation of a nucleophilic species **A**, which attacks the carbon atom of the C-Cl fragment, followed by the release of a nucleofuge species Cl^−^ and the formation of the target product (Scheme 2).

Structure of byspyrene-appended PVC **4** was confirmed by means of physical methods’ set. Thus, ^1^H NMR spectrum of polymer **4** contains resonance signals of the protons of the aromatic fragments of pyrene moieties as multiplets in a range of 7.46–8.06 ppm, resonance signals of the protons of aliphatic linkers as multiplets in the area of 5.54 ppm and resonance signals of the protons of PVC fragments as multiplets in the regions of 2.33–2.59 and 4.49–4.65 ppm. According to the ^1^H NMR data, the degree of modification of PVC with the pyrene fragment reaches 8%. The IR spectrum of polymer **4** contains absorption bands of the C=N (1648 cm^−1^), C-O (1254 cm^−1^), and C-H (2966 cm^−1^) groups, but no absorption band of the OH group (~3400 cm^−1^) of unreacted oxime. All of these facts indicate that the *O*-alkylation of the oxime moiety of **3** by C-Cl moieties of PVC core was successful. In addition, Gel Permeation Chromatography (GPC) experiments were carried out, and the number-average mass of the polymer **4** equal to M_n_ = 150 kDa was calculated with polydispersity index PDI = 1.3.

### 3.2. Photophysical Studies

As a next step, photophysical studies of compound **3** as well as polymer **4** were carried out. It worth mentioning, that the photophysical properties of the quite structurally similar compound, 1,5-di(pyren-1-yl)pentane, were studied and described by Zachariasse and Kühnle and us previously [15,28], so there was interest in comparison those with our current results.

**Figure 1:**
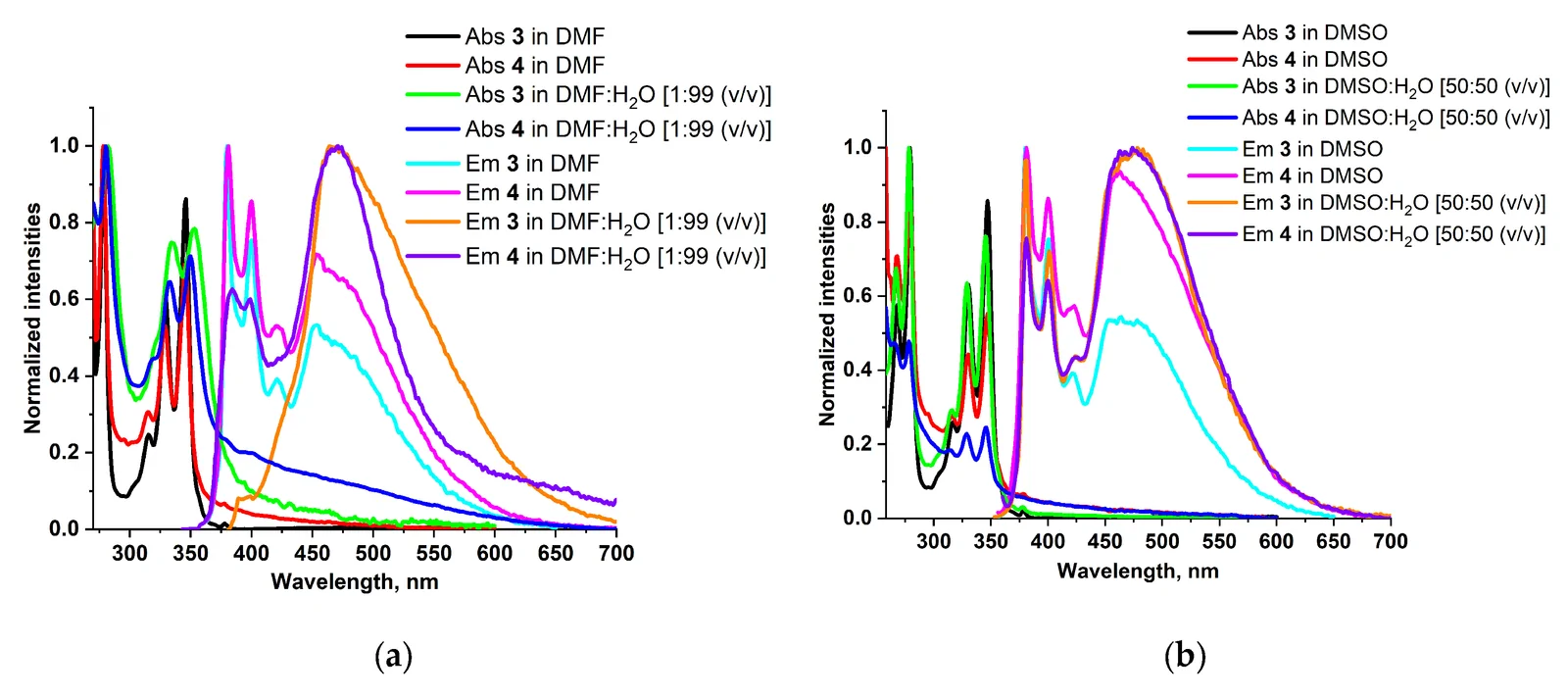
Normalized UV-Vis absorption and emission spectra of compounds **3**, **4** in DMF and DMF:H_2_O [1:99 (*v*/*v*)] (**a**) and DMSO and DMSO:H_2_O [50:50 (*v*/*v*)] (**b**).

It is no wonder, that the similar to pyrene and to 1,5-di(pyren-1-yl)pentane in particularly patterns were observed for compounds **3**, **4** (Figure 1, Table S1) as follows:

(1) The absorption spectra of compounds **3**, **4** in any media are indistinguishable from classic spectra of unsubstituted pyrene described in literature data and have characteristic maxima wavelength positions at ~328–335 nm (0-1) and 346–354 nm (0-0), attributed to S_0_ → S_2_ (π, π*) electronic transitions, as S_0_ → S_1_ (π, π*) one is forbidden for pyrene and its band located at ~378 nm have such low intensity, that it is barely noticeable [29]. In aqueous media absorption bands save their characteristic overall appearance, but became slightly red-shifted and broadened with less sharp vibronic structure (decrease of “peak-to-valley” ratio), also in DMF:H_2_O [1:99 (*v*/*v*)] absorption is also observed at wavelengths of 390 nm and above, where pyrene itself does not absorb—this indirectly indicates the possibility of the formation of pyrene dimers [30].
(2) As for the emission spectra the same pattern can be observed: characteristic for monomeric pyrene in its look and position emission band located in ~380–422 nm region, attributed to S_1_ → S_0_ electronic transition [29], which has clear-cut vibronic structure in purely organic media and is blurred in aqueous media. But difference from unsubstituted pyrene is in the presence of additional red-shifted (~452–474 nm) broad almost structureless band in all solvents/systems, which, as was already mentioned above, can be attributed to either formation of excimers or pre-associated pyrene dimers. The presence of not only monomeric, but also “excimeric” emission means, that our strategy of using bis-pyrene structure and attaching it as pendants to the flexible PVC backbone turned out to be successful.
(3) Depending on the media’s nature such pattern was observed: $\frac{I_{Ex}}{I_{M}}$ > 1 in aqueous media (it is 10.98 for **3** and 1.58 for **4** in DMF:H_2_O [1:99 (*v*/*v*)] and 1.03 for **3** and 1.31 for **4** in DMSO:H_2_O [50:50 (*v*/*v*)]), while it’s the opposite—$\frac{I_{Ex}}{I_{M}}$ < 1—in pure solvents (0.53 for **3** and 0.72 for **4** in DMF and 0.54 for **3** and 0.93 for **4** in DMSO); where I_Ex_ is the intensity of the “excimeric” emission and I_M_ is the intensity of the monomeric one.
(4) No particular differences in photophysical properties/spectral data between compound **3** and its polymeric derivative **4** were found.

**Figure 2:**
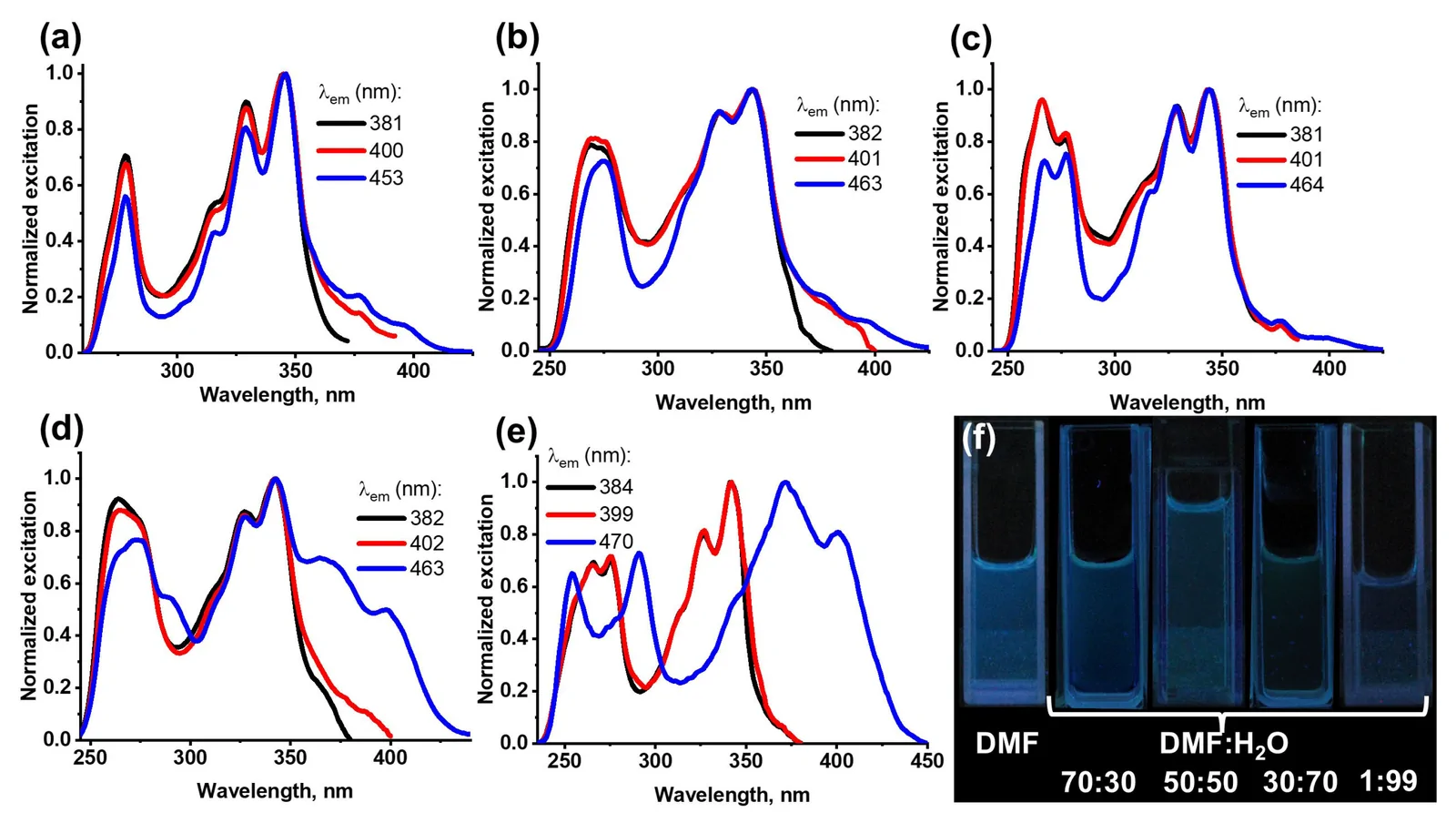
Excitation spectra of polymer **4** recorded at different wavelengths in DMF (**a**), DMF:H_2_O [70:30 (*v*/*v*)] (**b**), DMF:H_2_O [50:50 (*v*/*v*)] (**c**), DMF:H_2_O [30:70 (*v*/*v*)] (**d**) and DMF:H_2_O [1:99 (*v*/*v*)] (**e**); photographs of polymer **4** in different media taken under UV lamp (λ_Ex_ = 354 nm) (**f**).

To study the exact nature of a broad-band longer-wavelength emission, the excitation spectra were recorded at the wavelengths of the monomer and excimer maxima in different media (Figure 2). Whereas in pure DMF, DMF:H_2_O [70:30 (*v*/*v*)] and in DMF:H_2_O [50:50 (*v*/*v*)] the spectra recorded at the wavelengths of the monomer and excimer maxima were almost coincide with each other (Figure 2a–c), in DMF:H_2_O [30:70 (*v*/*v*)] and DMF:H_2_O [1:99 (*v*/*v*)] the excitation spectra recorded at the wavelengths of the excimer maxima were distinguished by the presence of characteristic signs of aggregation, already described earlier for the corresponding absorption spectra: broadening, bathochromic shift and a decrease in the “peak-to-valley” ratio (Figure 2d,e) [31].

On the photos below (Figure 2f) it can be seen, that emission intensity decreases simultaneously with increase of water content, which indicates the occurrence of ACQ.

### 3.3. Fluorometric Titration Experiments

**Figure 3:**
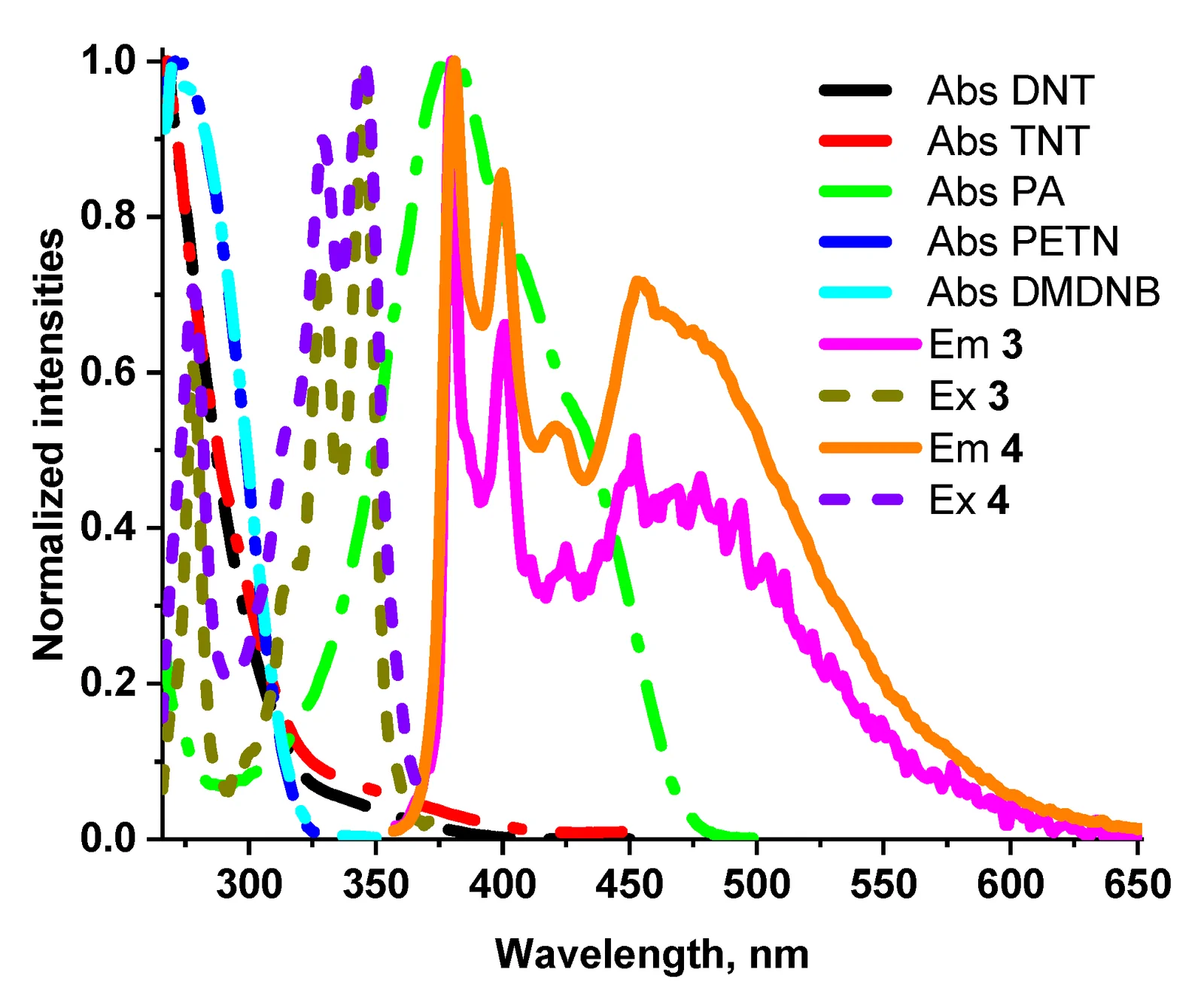
An overlay spectra of absorption (Abs) of DNT, TNT, PA, PETN, DMDNB and emission (Em) and excitation (Ex) of compounds **3**, **4** in DMF.

To explore a sensory property towards explosive nitro-compounds and possible practical application of obtained compounds, especially for the polymer **4**, fluorometric titration studies in the presence of 2,4-dinitrotoluene (DNT), 2,4,6-trinitrotoluene (TNT), 2,3-dimethyl-2,3-dinitrobutane (DMDNB), pentaerythritol tetranitrate (PETN) and 2,4,6-trinitrophenol (picric acid, PA) were carried out in DMF. It should be mentioned, that the possibility of Inner Filter Effect (IFE) was taken into account, due to the fact that absorption spectra of some nitro-analytes are overlapped with excitation and emission spectra of compounds **3**, **4** (Figure 3), the correction of obtained titration results were also carried out. The obtained corrected data are summarized in Table 1.

Interestingly, despite the fact, that there were no differences in photophysical properties of compounds **3**, **4**, their sensory properties are differed significantly.

**Figure 4:**
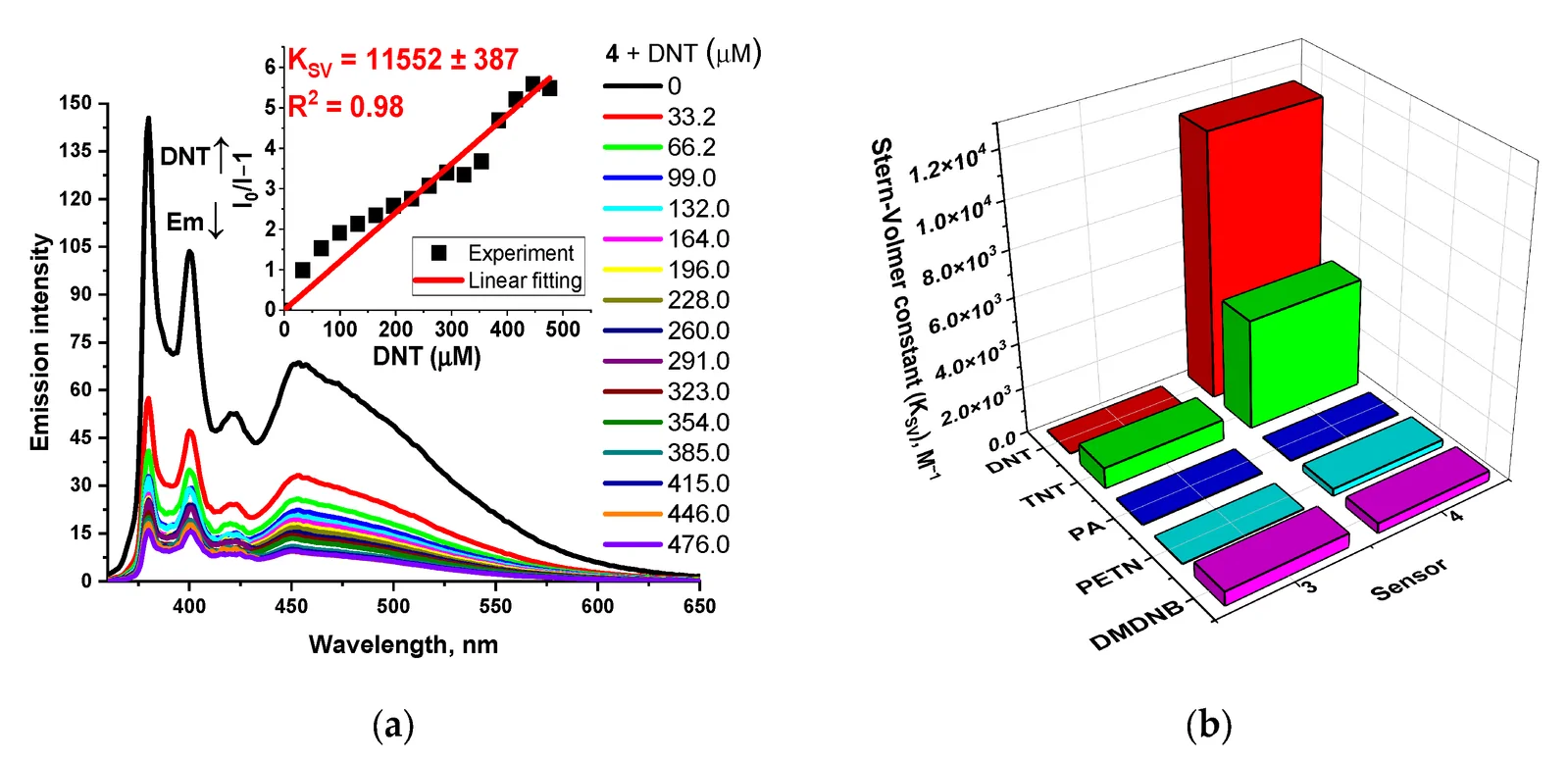
Fluorescence quenching of polymer **4** in the presence of DNT (**a**), insert—Stern-Volmer plot of the fluorescence “*turn-off*” response of **4** to DNT after inner filter effect (IFE) correction; 3D bar graph of the Stern-Volmer constant values (**b**) for compounds **3**, **4** in the presence of various nitro-containing analytes.

Thus, in case of polymer **4** a dramatic quenching of fluorescence took place upon the addition of nitro-aromatic DNT (Figure 4a) and TNT, whereas only slight fluorescence “*turn-off*” response was observed in the presence of nitro-aliphatic PETN and DMDNB (Figure 4b, see also Supplementary Information). On the other hand, compound **3** gave a weak, almost neglectable, fluorescence “*turn-off*” response only in the presence of TNT and DMDNB. For the both compounds **3**, **4** the fluorescence quenching upon addition of PA was observed, but it was attributed exclusively to a double IFE (Table 1). This phenomenon is expected, considering the high absorbance of PA both at excitation and emission wavelength (Figure 3). High linearity of Stern-Volmer fluorescence quenching plots can be attributed to the static quenching mechanism.

In our opinion, such a pronounced difference in sensory properties can be explained by a higher concentration/count of bispyrene units in the polymer **4**, leading to formation of local accumulated “excimer pockets/blobs”, which increases the probability of successful diffusion of nitro-analytes and their effective collision with pyrenes. These interactions make quenching of these PVC-attached excimers easer compared to the single molecules of compound **3** of low molar weight bearing onlytwo pyrene units linked with each other, and these molecules are diffusely distributed across the DMF solution.

It is also clear, that polymer **4** has higher selectivity towards DNT, compared to TNT (Stern-Volmer constants were calculated to be equal to 1.15 × 10^4^ for DNT and 4.82 × 10^3^ for TNT (Table 1)). Probably, it can be attributed to the lesser affinity of the TNT to the PVC as well as higher steric hindrance caused by a bulkier TNT molecular structure, which makes the diffusion process into the polymer **4** more difficult. And we have observed and described already the same phenomenon for some water-soluble pyrene derivatives previously [32].

We have stipulated previously that the solution environment can be the driving force for the diffusion of analytes to the environment of the sensor—for example, in the case of poorly water soluble DNT and TNT [33] the aqueous media may promote the migration of these analytes to a more “friendly” in nature non-polar PAH units [9,15,32]. Eager to explore such possibility for the polymer **4** we carried out fluorometric titration experiments in aqueous media with different water content, in particularly, in DMF:H_2_O [70:30 (*v*/*v*)], DMF:H_2_O [50:50 (*v*/*v*)], DMF:H_2_O [30:70 (*v*/*v*)] and DMF:H_2_O [1:99 (*v*/*v*)]. Thus, only in DMF:H_2_O [70:30 (*v*/*v*)] some of the sensory properties of polymer **4** were observed, but the Stern-Volmer constant value for DNT was calculated to be pretty low (1.58 × 10^3^ M^−1^, see Figure S21). And no fluorescence quenching was observed in both media in the presence of any nitro-analytes.

It can be suggested, that the polymer chain folds up into a dense globule in an unfavorable, “non-friendly” aqueous environment for its non-polar lipophilic structure which hinders diffusion of nitro-analytes to the tightly packed, aggregated pyrene units, thus making fluorescence quenching difficult or impossible.

Sometimes aggregates have elevated sensitivity to analytes and such examples have been described in the literature data [31], but in majority of cases aggregation is undesirable phenomenon in chemosensing due to both ACQ and diffusion hindrance, which is clearly can be seen in case of polymer **4**.

It is clear that polymer **4** is on par with majority of them in terms of quenching constants values and quenching efficiency as it can be seen from comparing sensory properties of polymer **4** with other previously described PAH-based polymeric materials (Table 2). The advantages of polymer **4** over the given examples are both in the simplicity of the PVC modifier’s structure **3** as well as in the ease and environmental friendliness of the polymer **4** synthesis. Utilizing of PVC as a backbone is also beneficial, as it is a common waste material, and the possibility of turning it into efficient but cheap sensory material corresponds perfectly to the “from waste to wealth” concept, which is currently trending.

## 4. Conclusions

In summary, PVC-based bis-pyrene-containing polymeric sensory material was prepared for a first time *via* mechanosynthesis under ball-milling conditions and its sensory properties towards common nitro-explosives such as 2,4-dinitrotoluene, 2,4,6-trinitrotoluene, picric acid, 2,3-dimethyl-2,3-dinitrobutane and pentaerythritol tetranitrate were studied. The herein reported bola-type bis-pyrene appended PVC exhibited a profound fluorescence “*turn-off*” response with up to 10^4^ M^−1^ quenching constants in the presence of nitro-aromatic analytes (DNT and TNT) in DMF media which is on par with those described in the literature for other PAH-based polymeric sensory materials. Some selectiveness for DNT vs. TNT was observed as well.

## Figures and Tables

**Table 1:** Stern-Volmer constants (K_SV_), Quenching Efficiency (QE) and Limit of Detection (LOD) of compounds **3**, **4** in the presence of various nitro-containing analytes in DMF.

Quantity	Analyte	Compound			
		3		4	
		Before ^1^	After ^1^	Before ^1^	After ^1^
**K_SV_ (M** ^−**1**^ **)**	**DNT**	0.67 × 10^3^	n.a. ^2^	1.30 × 10^4^	1.15 × 10^4^
	**TNT**	1.75 × 10^3^	0.90 × 10^3^	6.58 × 10^3^	4.82 × 10^3^
	**PA**	2.71 × 10^3^	n.a. ^3^	7.74 × 10^3^	n.a. ^3^
	**PETN**	n.a. ^4^	n.a. ^4^	0.30 × 10^3^	0.30 × 10^3^
	**DMDNB**	0.63 × 10^3^	0.61 × 10^3^	0.43 × 10^3^	0.43 × 10^3^
**QE (%)**	**DNT**	21	n.a. ^2^	86	85
	**TNT**	45	28	77	70
	**PA**	15	n.a. ^3^	28	n.a. ^3^
	**PETN**	n.a. ^4^	n.a. ^4^	14	14
	**DMDNB**	19	19	17	17
**LOD (M)**	**DNT**	n.a. ^5^	n.a. ^2^	n.a. ^5^	5.41 × 10^−4^
	**TNT**	n.a. ^5^	1.60 × 10^−4^	n.a. ^5^	1.19 × 10^−4^
	**PA**	n.a. ^5^	n.a. ^3^	n.a. ^5^	n.a. ^3^
	**PETN**	n.a. ^5^	n.a. ^4^	n.a. ^5^	2.38 × 10^−4^
	**DMDNB**	n.a. ^5^	2.94 × 10^−4^	n.a. ^5^	1.13 × 10^−3^

^1^ Before and after inner filter effect (IFE) correction; ^2^ Low linearity (R^2^ < 0.95); ^3^ Fluorescence quenching was attributed purely to IFE; ^4^ No sensory response; ^5^ The value was not calculated.

**Table 2:** Comparison of various PAH-based polymeric materials for the detection of nitro-containing analytes.

Compounds		K_SV_ (M^−1^)	QE (%)	LOD	Solvent	Ref.
**P(PySt-EGDMA) microbeads**		1.4 × 10^6^ (DNT)	n.d.	7.63 × 10^−9^ M (DNT)	Water	[34]
		2.2 × 10^5^ (TNT)		8.81 × 10^−8^ M (TNT)		
		2.9 × 10^4^ (PA)		1.51 × 10^−6^ M (PA)		
		5.2 × 10^4^ (NP)		1.05 × 10^−6^ M (NP)		
		2.2 × 10^5^ (NB)		1.11 × 10^−7^ M (NB)		
		9.0 × 10^3^ (NE)		3.91 × 10^−6^ M (NE)		
		3.7 × 10^3^ (DNT)	n.d.	1.28 × 10^−6^ M (DNT)	Methanol	
		5.9 × 10^3^ (TNT)		1.74 × 10^−6^ M (TNT)		
		2.4 × 10^4^ (PA)		1.02 × 10^−6^ M (PA)		
		2.1 × 10^3^ (DNT)	n.d.	3.15 × 10^−6^ M (DNT)	MeCN	
		2.1 × 10^3^ (TNT)		1.32 × 10^−6^ M (TNT)		
		1.6 × 10^4^ (PA)		3.29 × 10^−7^ M (PA)		
		7.1 × 10^3^ (NP)		9.35 × 10^−7^ M (NP)		
		1.7 × 10^3^ (NB)		3.52 × 10^−6^ M (NB)		
		1.6 × 10^3^ (NE)		5.72 × 10^−6^ M (NE)		
		1.4 × 10^3^ (DNT)	n.d.	5.76 × 10^−6^ M (DNT)	THF	
		0.6 × 10^3^ (TNT)		6.53 × 10^−6^ M (TNT)		
		8.8 × 10^3^ (PA)		9.26 × 10^−7^ M (PA)		
**Polymer films**	**PB**	3.1 × 10^4^ (DNT)	n.d.	n.d.	Water	[13]
		1.5 × 10^5^ (TNT)				
		1.6 × 10^4^ (NT)				
		3.9 × 10^3^ (NB)				
		1.2 × 10^4^ (PA)				
	**PA**	2.3 × 10^4^ (TNT)	n.d.	n.d.		
	**PC**	3.8 × 10^4^ (TNT)	n.d.	n.d.		
	**PE**	2.0 × 10^4^ (TNT)	n.d.	n.d.		
	**PF**	1.9 × 10^4^ (TNT)	n.d.	n.d.		
**Poly(PHP-*alt*-3HT)**		1.1 × 10^4^ (TNT)	78 (TNT)	4.67 μM (TNT)	THF	[35]
**Poly(PHP-*r*-3HT-*r*-BTz)**		1.6 × 10^4^ (TNT)	81 (TNT)	2.01 μM (TNT)		
**P2**		1.5 × 10^5^ (TNT)	72 (TNT)	64 nM (TNT)	Water	[36]
**Py-3T-functionalized film**		3.72 × 10^4^ (PA)	70 (PA)	2.08 × 10^−7^ mol/L (PA)	Water	[37]
**P(LA3-co-ADTC1-pyrene)**		2.37 × 10^3^ (PA)	94 (PA)	n.d.	THF:water (3:1)	[38]
**Compounds 1–6**		8.1 × 10^3^–3.8 × 10^4^ (PA)	n.d.	n.d.	CHCl_3_	[39]
**Film 1 (PyPE-1)**		3.62 × 10^3^ (DNT)	n.d.	n.d.	Water	[40]
		3.26 × 10^4^ (TNT)				
		1.34 × 10^3^ (NB)				
		0.45 × 10^3^ (PA)				
**Film 2 (PyPE-2)**		3.65 × 10^4^ (TNT)	n.d.	n.d.		
**[DPyDBzPBI-BuI][Br]**		2.50 × 10^4^ (TNT)	60 (TNT)	n.d.	DMSO	[11]
		1.78 × 10^4^ (NB)	46 (NB)			
		4.15 × 10^4^ (PA)	70 (PA)			
**[DAnDBzPBI-BuI][Br]**		1.88 × 10^4^ (TNT)	53 (TNT)	n.d.		
		1.59 × 10^4^ (NB)	44 (NB)			
		2.03 × 10^4^ (PA)	55 (PA)			
**Py−PES nanofiber film**		1.26 × 10^6^ (PA)	n.d.	23 nM (PA)	Water	[12]
		1.80 × 10^5^ (TNT)		160 nM (TNT)		
		7.52 × 10^4^ (DNT)		400 nM (DNT)		
		3.26 × 10^4^ (RDX)		980 nM (RDX)		
**P**		5.96 × 10^4^ (DNT)	n.d.	1.35 × 10^−6^ M (DNT)	THF	[41]
		13.57 × 10^4^ (TNT)		9.39 × 10^−7^ M (TNT)		
		0.56 × 10^4^ (NB)		1.06 × 10^−5^ M (NB)		
		15.86 × 10^4^ (PA)		5.78 × 10^−7^ M (PA)		
**Polymer 4**		1.15 × 10^4^ (DNT)	85 (DNT)	5.41 × 10^−4^ M (DNT)	DMF	Present work
		4.82 × 10^3^ (TNT)	70 (TNT)	1.19 × 10^−4^ M (TNT)		

## Data Availability

Data are contained within article and Supporting Information.

## Supplementary Materials

The following supporting information can be downloaded at https://www.mdpi.com/article/10.3390/s26175342/s1, Figure S1: NMR ^1^H spectra of compound **2** 400 MHz, CDCl_3_; Figure S2: NMR ^1^H spectra of compound **3** 400 MHz, DMSO-d_6_; Figure S3: NMR ^1^H spectra of compound **3** 400 MHz, DMF-d_7_; Figure S4: NMR ^1^H spectra of compound **4** 400 MHz, DMF-d_7_; Figure S5: NMR ^13^C spectra of compound **2** 150 MHz, DMSO-d_6_; Figure S6: NMR ^13^C spectra of compound **3** 150 MHz, DMSO-d_6_; Figure S7: IR (DRA) spectrum of compound **2**; Figure S8: IR (DRA) spectrum of compound **3**; Figure S9: IR (DRA) spectrum of compound **4**; Figure S10: HRMS (ESI) spectrum of compound **2**; Figure S11: HRMS (ESI) spectrum of compound **3**; Table S1: Summary of photophysical data for compounds **3, 4**; Table S2: Excitation and emission wavelengths of compounds **3, 4** in fluorescence titration experiments in DMF and K_SV_ values and IFE correction calculations; Figure S12: Overlayed spectra of fluorescence quenching of **3** upon addition of DNT in DMF; Figure S13: Overlayed spectra of fluorescence quenching of **3** upon addition of TNT in DMF; insert—Stern-Volmer plot of the fluorescence “*turn-off*” response of **3** to TNT after inner filter effect (IFE) correction; Figure S14: Overlayed spectra of fluorescence quenching of **3** upon addition of PA in DMF; Figure S15: Overlayed spectra of fluorescence quenching of **3** upon addition of DMDNB in DMF; insert—Stern-Volmer plot of the fluorescence “*turn-off*” response of **3** to DMDNB after inner filter effect (IFE) correction; Figure S16: Overlayed spectra of fluorescence quenching of **4** upon addition of TNT in DMF; insert—Stern-Volmer plot of the fluorescence “*turn-off*” response of **4** to TNT after inner filter effect (IFE) correction; Figure S17: Overlayed spectra of fluorescence quenching of **4** upon addition of PA in DMF; Figure S18: Overlayed spectra of fluorescence quenching of **4** upon addition of PETN in DMF; insert—Stern-Volmer plot of the fluorescence “*turn-off*” response of **4** to PETN after inner filter effect (IFE) correction; Figure S19: Overlayed spectra of fluorescence quenching of **4** upon addition of DMDNB in DMF; insert—Stern-Volmer plot of the fluorescence “*turn-off*” response of **4** to DMDNB after inner filter effect (IFE) correction; Figure S20: Normalized UV-Vis absorption and emission spectra of polymer **4** in DMF:H_2_O [70:30 (*v*/*v*)], DMF:H2O [50:50 (*v*/*v*)] and DMF:H_2_O [30:70 (*v*/*v*)]; Figure S21: Overlayed spectra of fluorescence quenching of **4** upon addition of DNT in DMF:H_2_O [70:30 (*v*/*v*)]; insert—Stern-Volmer plot of the fluorescence “*turn-off*” response of **4** to DNT after inner filter effect (IFE) correction; Figure S22: Overlayed spectra of fluorescence quenching of **4** upon addition of DNT in DMF (left); Stern-Volmer plot of the fluorescence “*turn-off*” response of **4** to DNT after inner filter effect (IFE) correction (right). The first parallel; Figure S23: Overlayed spectra of fluorescence quenching of **4** upon addition of DNT in DMF (left); Stern-Volmer plot of the fluorescence “*turn-off*” response of **4** to DNT after inner filter effect (IFE) correction (right). The second parallel; Figure S24: Overlayed spectra of fluorescence quenching of **4** upon addition of DNT in DMF (left); Stern-Volmer plot of the fluorescence “*turn-off*” response of **4** to DNT after inner filter effect (IFE) correction (right). The third parallel.

## Abbreviations

The following abbreviations are used in this manuscript:

ACQ	Aggregation-Caused Quenching
DMDNB	2,3-Dimethyl-2,3-dinitrobutane
DNT	2,4-Dinitrotoluene
IFE	Inner Filter Effect
LOD	Limit Of Detection
PA	2,4,6-Trinitrophenol, picric acid
PAH	Polycyclic Aromatic Hydrocarbons
PETN	Pentaerythritol tetranitrate
PVC	Poly Vinyl Chloride
QE	Quenching Efficiency
TNT	2,4,6-Trinitrotoluene
