# Supplementary material for: Bis-Pyrene-Appended Polyvinyl Chloride: Mechanosynthesis and Nitro-Explosive Detection
Source: Sensors (Basel). 2026 Aug 24;26(17):5342. doi: 10.3390/s26175342 (PMC13568286; doi:10.3390/s26175342)
Supplement: Supplementary file 1 [file sensors-26-05342-s001.zip › sensors-4451728-supplementary.pdf]

Supporting info

Bis-Pyrene-Appended Polyvinyl Chloride: Mechanosynthesis and Nitro-Explosive Detection

Vadim A. Platonov [1,2], Leila K. Sadieva [1,*], Mikhail. A. Makovskiy [1], Nikolay A. Belyaev [1], Tatiana I. Shendrikova [1,2], Artem V. Baklykov [1,2], Dmitry S. Kopchuk [1,2], Igor S. Kovalev [1], Grigory V. Zyryanov [1,2] and Valeriy N. Charushin [1,2]

1 Institute of Chemical Engineering, Ural Federal University, 19, Mira Str., Yekaterinburg 620002, Russia; vplatonov10@mail.ru (V.A.P.); ma-makovsy@mail.ru (M.A.M.); n.a.beliaev@urfu.ru (N.A.B.); t.i.shenderikova@urfu.ru (T.I.S.); art.baklykov@gmail.com (A.V.B.); dkopchuk@mail.ru (D.S.K.); ekls85@yandex.ru (I.S.K.); g.v.zyrianov@urfu.ru (G.V.Z.); charushin@ios.uran.ru (V.N.C.)

2 I. Ya. Postovsky Institute of Organic Synthesis of RAS (Ural Division), 22/20, S. Kovalevskoy/Akademicheskaya Str., Yekaterinburg 620137, Russia

* Correspondence: l.k.sadieva@urfu.ru

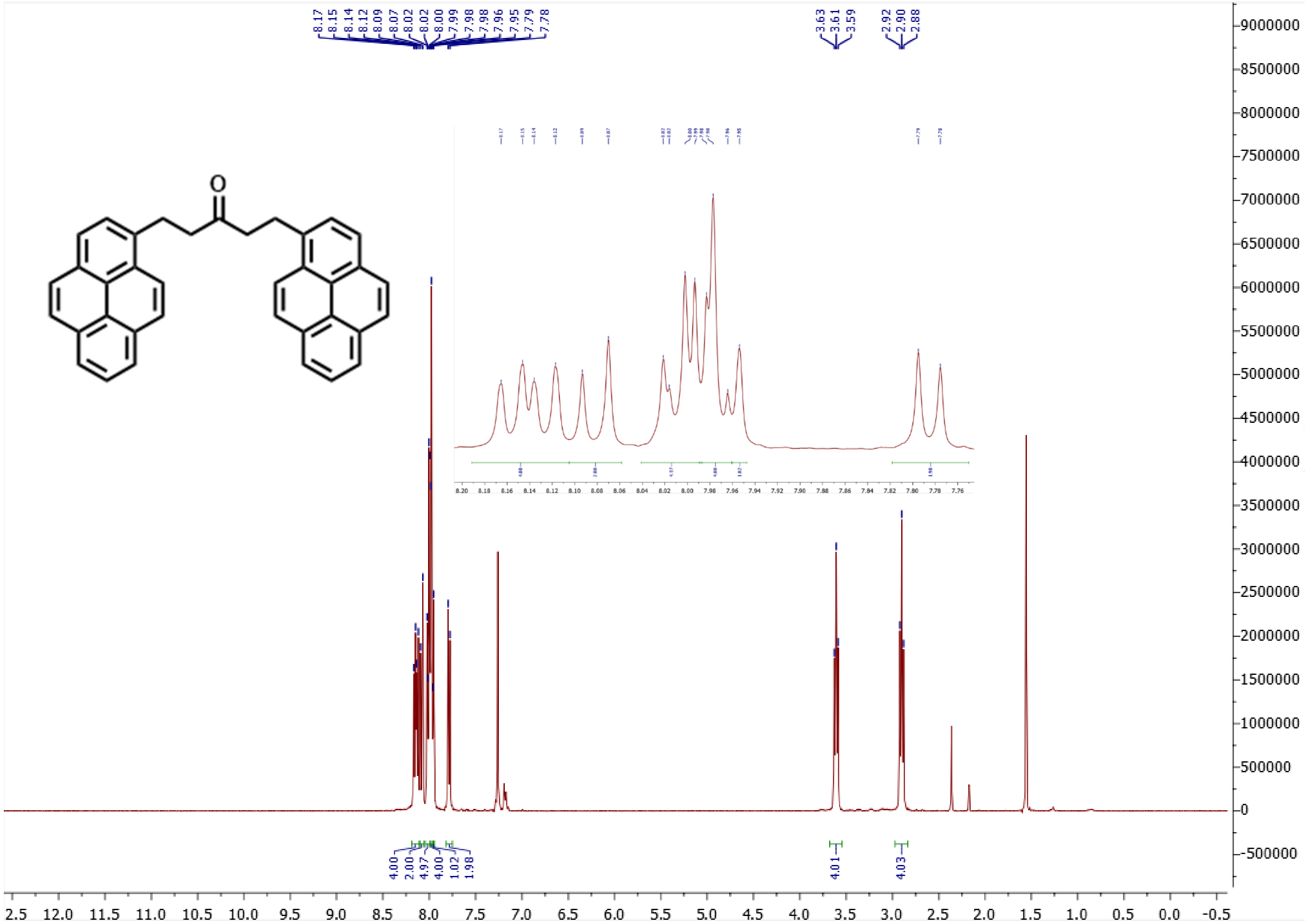

Figure S1. NMR ^{1}H spectra of compound **2** 400 MHz, $CDCl_3$

Supporting info

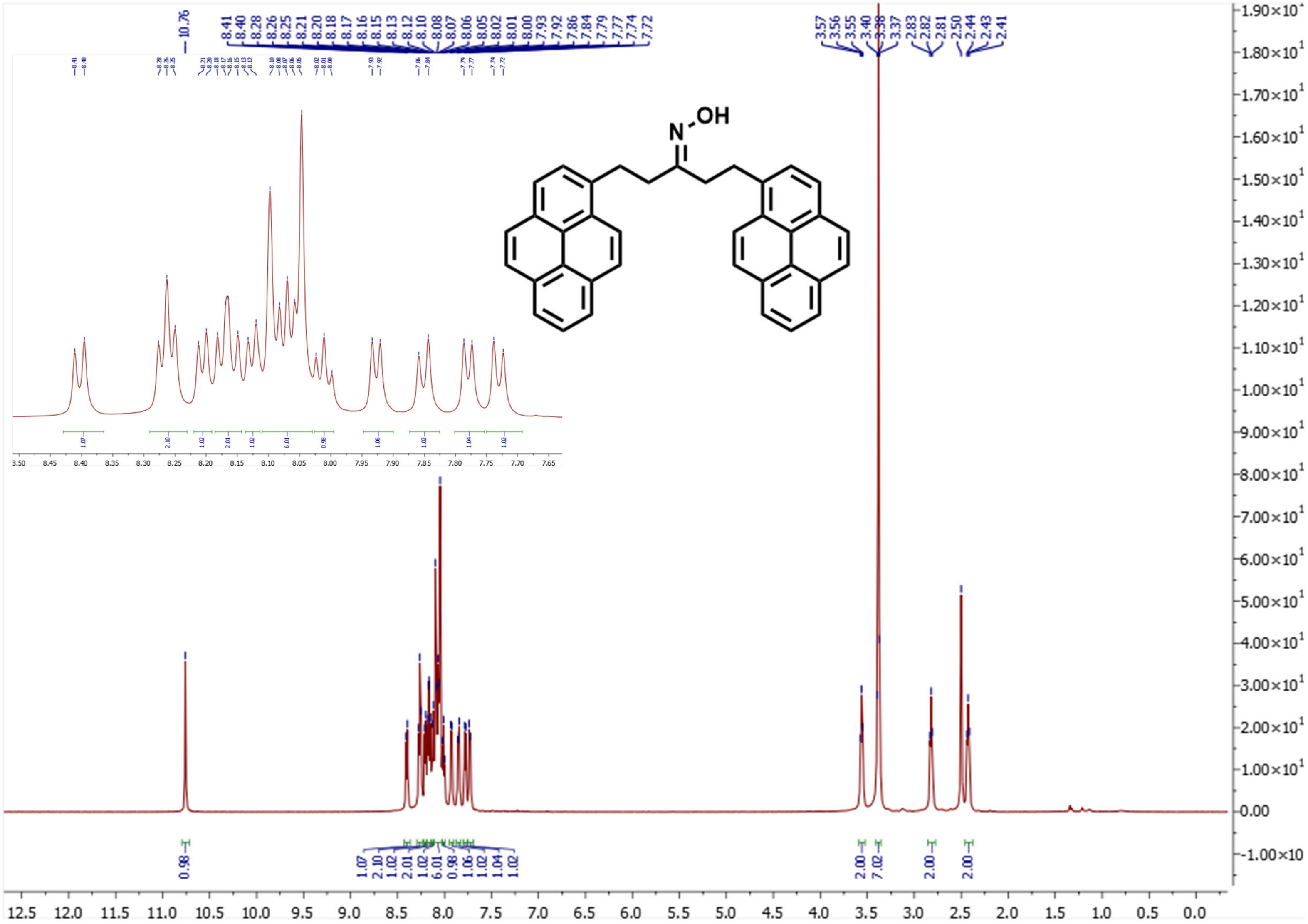

Figure S2. NMR ^{1}H spectra of compound **3** 400 MHz, DMSO-d_6

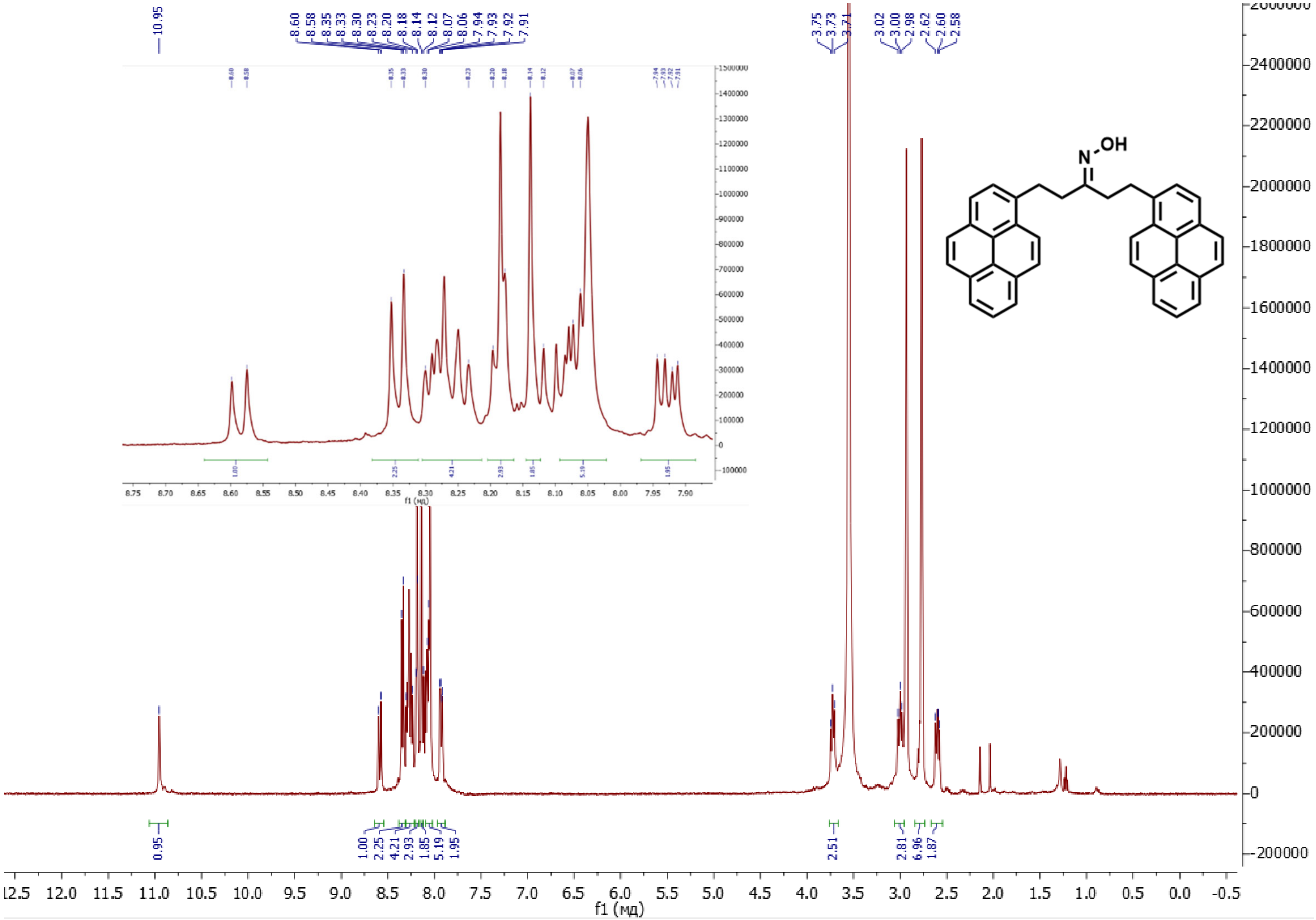

Figure S3. NMR ^{1}H spectra of compound **3** 400 MHz, DMF-d_7

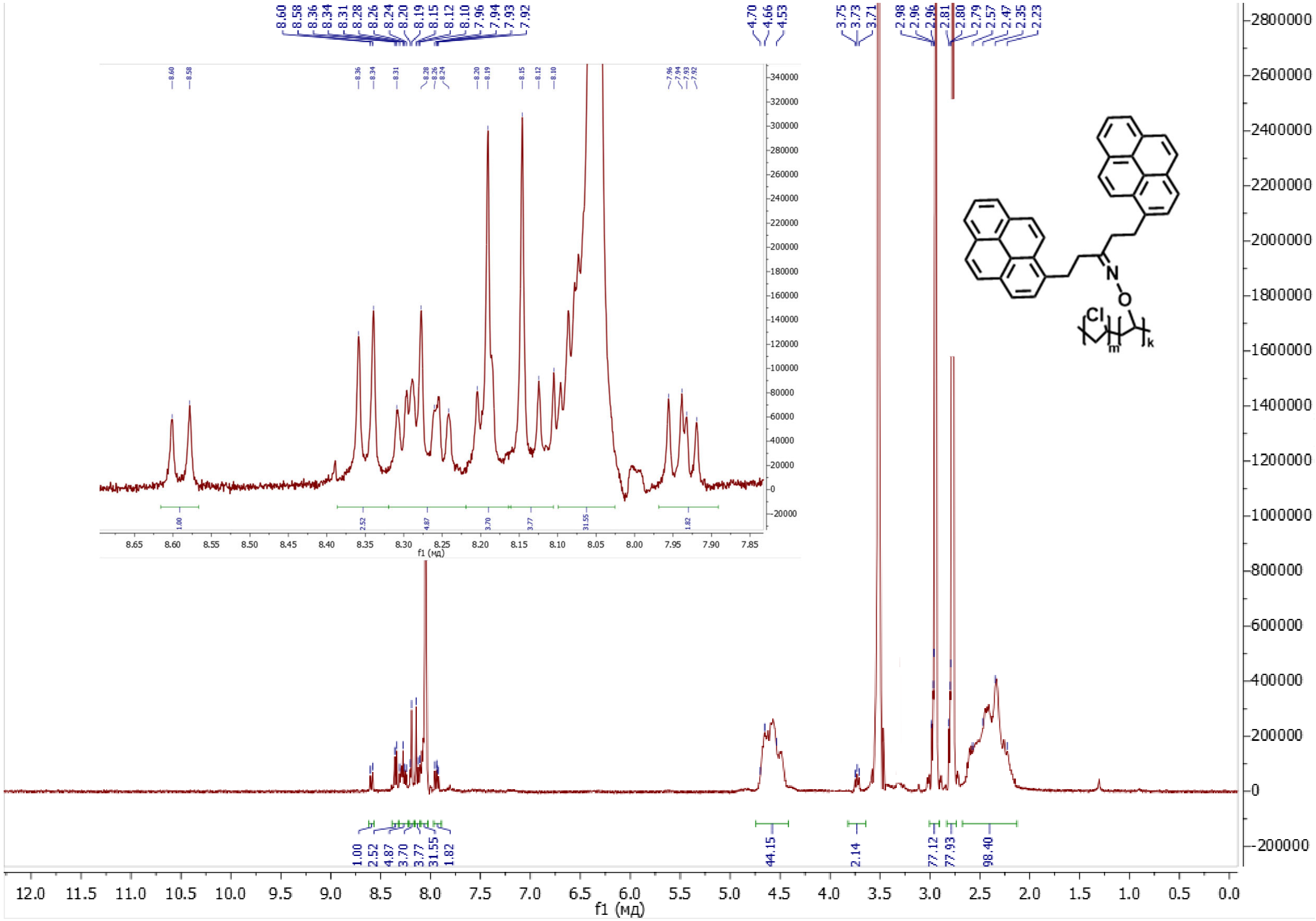

Figure S4. NMR ^{1}H spectra of compound **4** 400 MHz, DMF-d_7

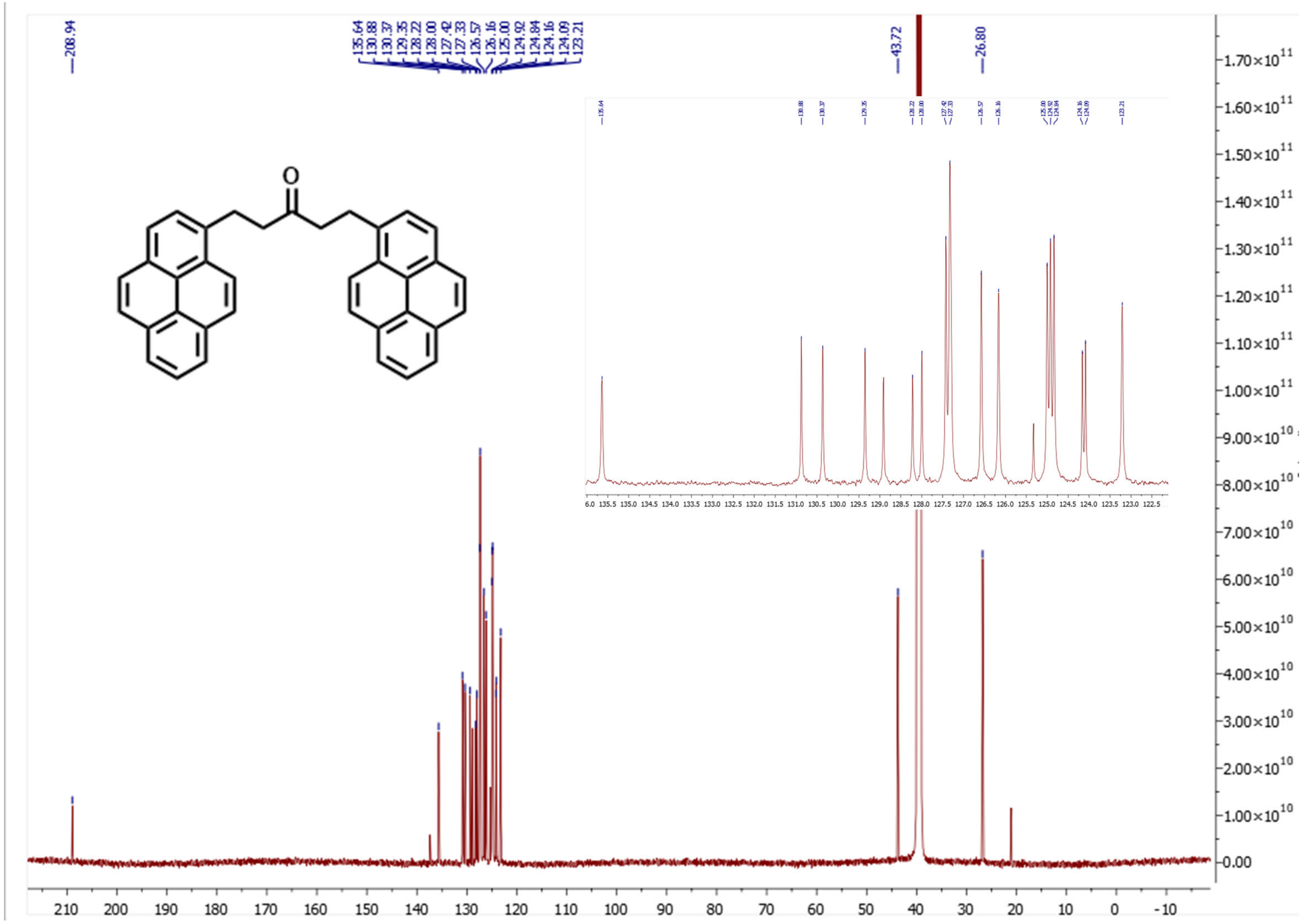

Figure S5. NMR ^{13}C spectra of compound **2** 150 MHz, DMSO-d_6

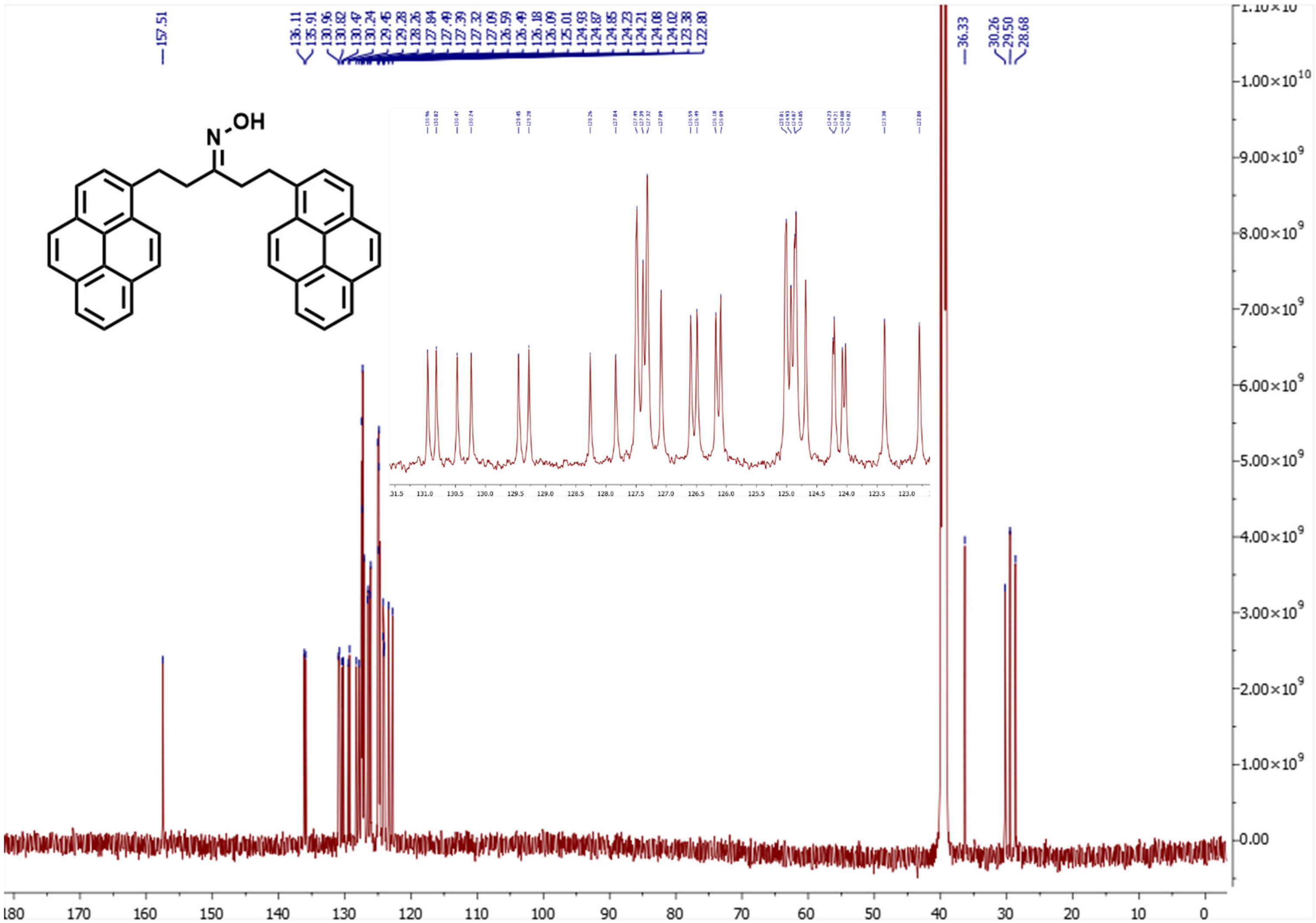

Figure S6. NMR ^{13}C spectra of compound **3** 150 MHz, DMSO-d_6

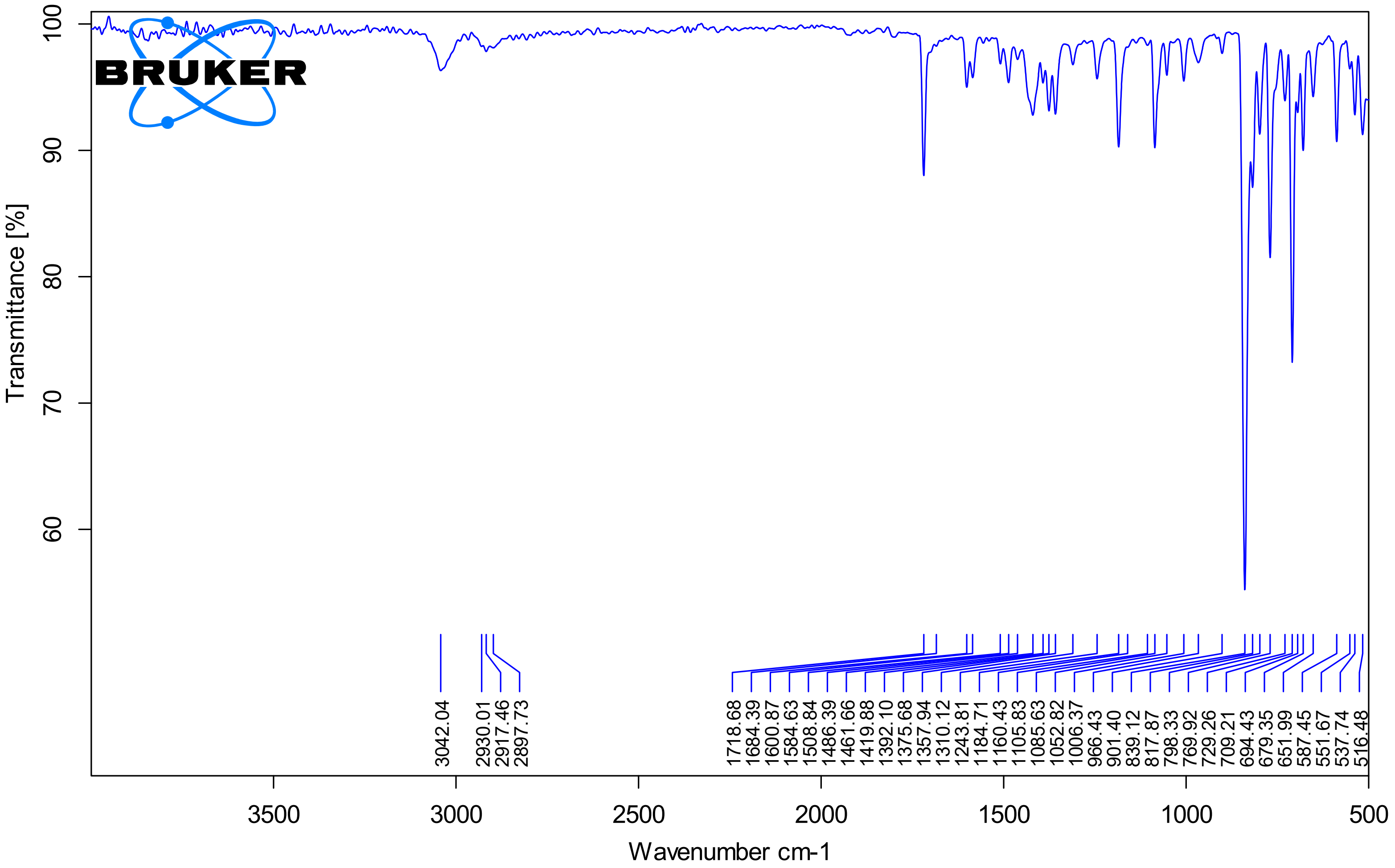

Figure S7. IR (DRA) spectrum of compound **2**

Supporting info

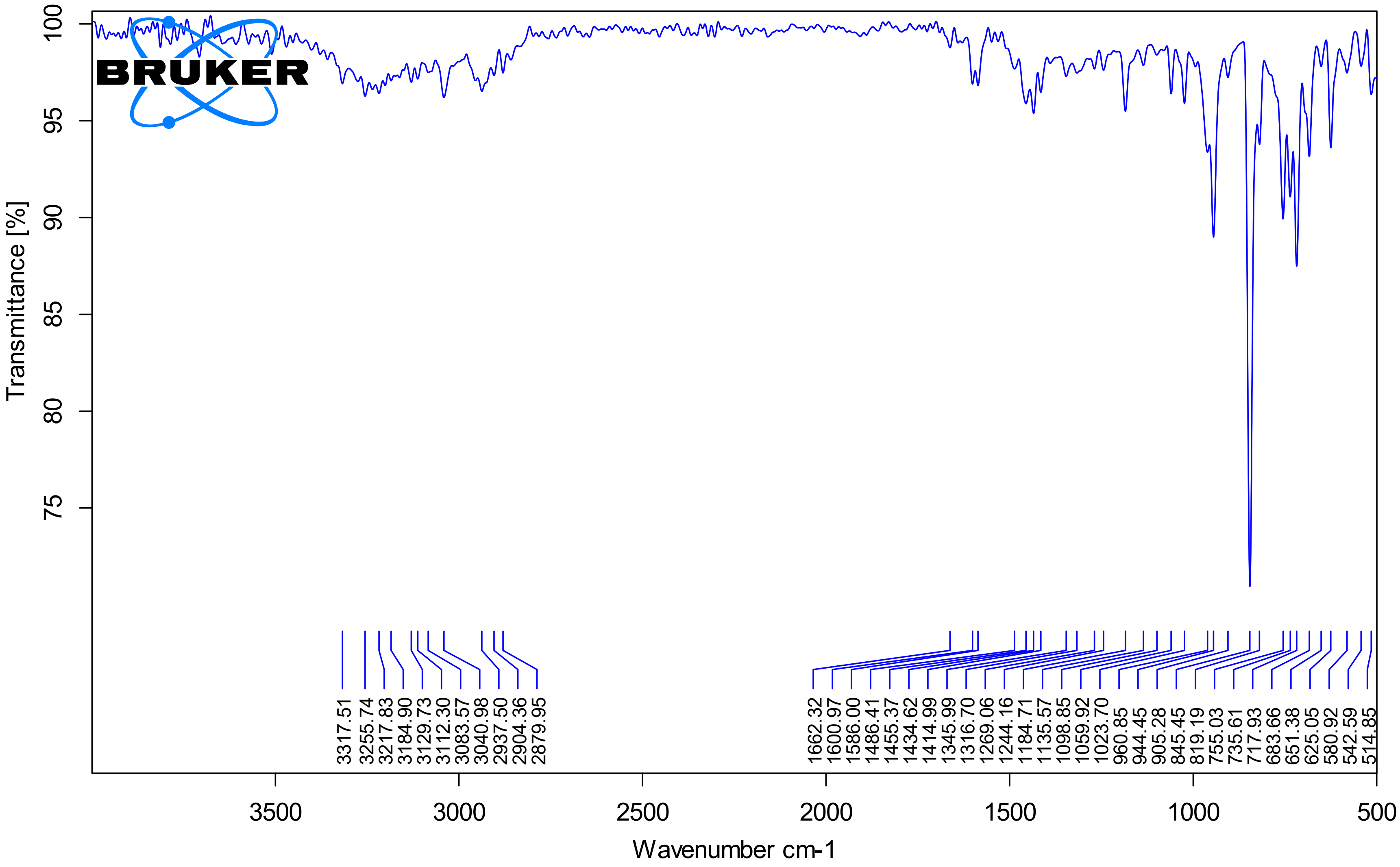

Figure S8. IR (DRA) spectrum of compound **3**

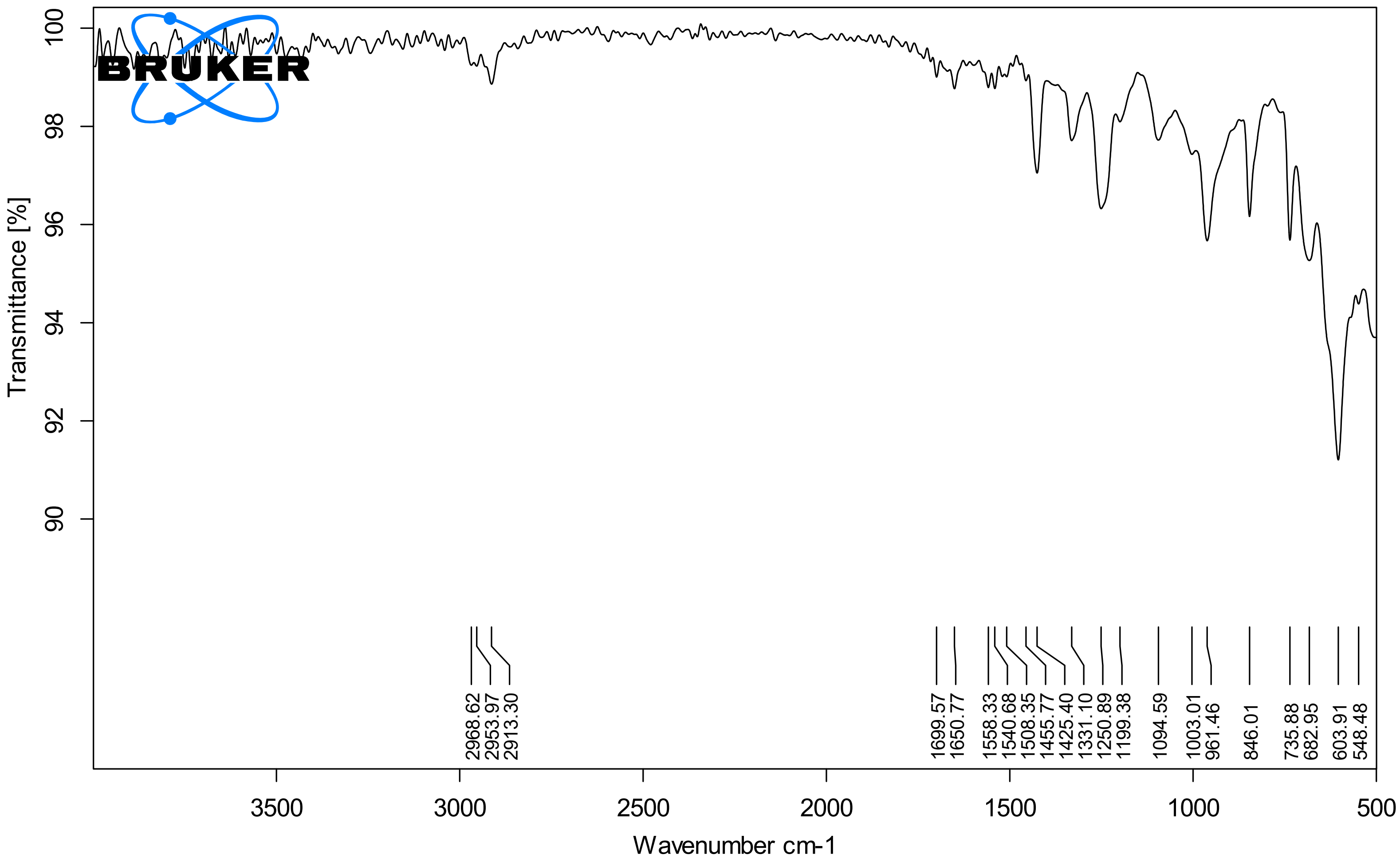

Figure S9. IR (DRA) spectrum of compound **4**

Qualitative Compound Report

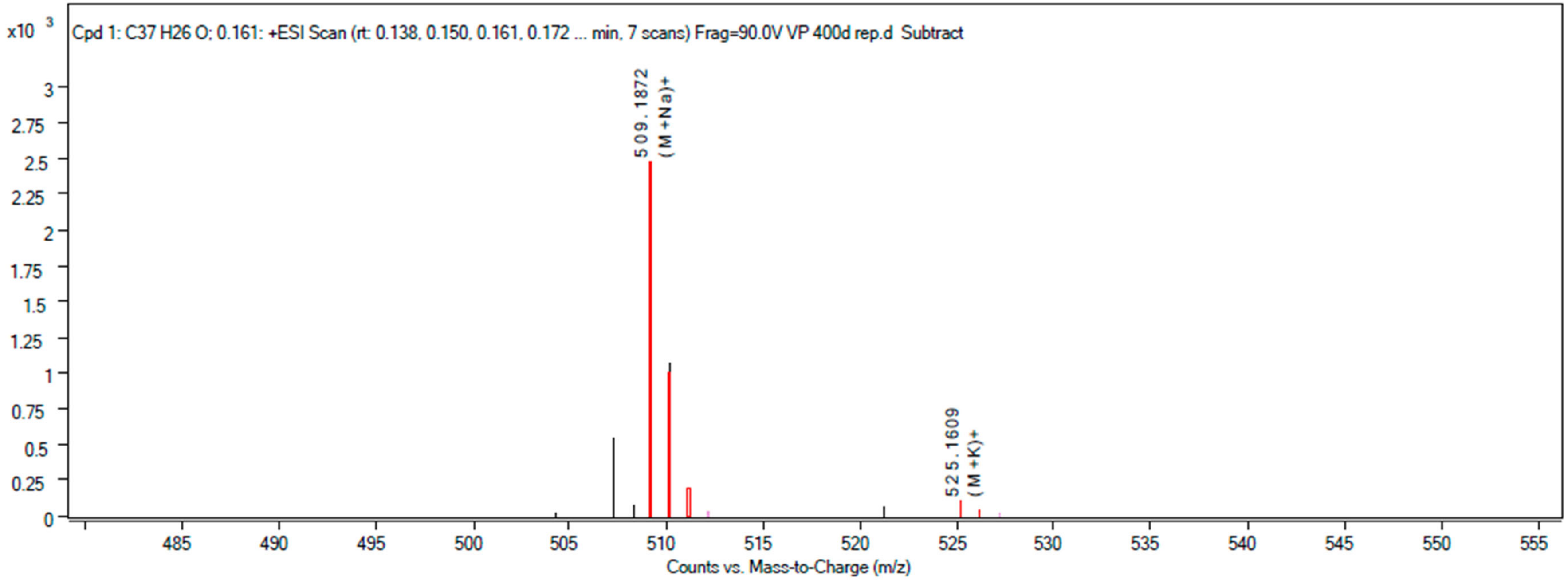

MS Spectrum Peak List

m/z	Calc *m/z*	Diff(ppm)	z	Abund	Ion
509.1872	509.1876	0.74	1	2479.85	(M+Na)+
510.1902	510.191	1.44	1	1063.71	(M+Na)+
511.1946	511.1943	-0.64	1	130.75	(M+Na)+
525.1609	525.1615	1.23	1	112.41	(M+K)+
526.1675	526.1649	-4.98	1	29.87	(M+K)+

--- End Of Report ---

Figure S10. HRMS (ESI) spectrum of compound **2**

Qualitative Compound Report

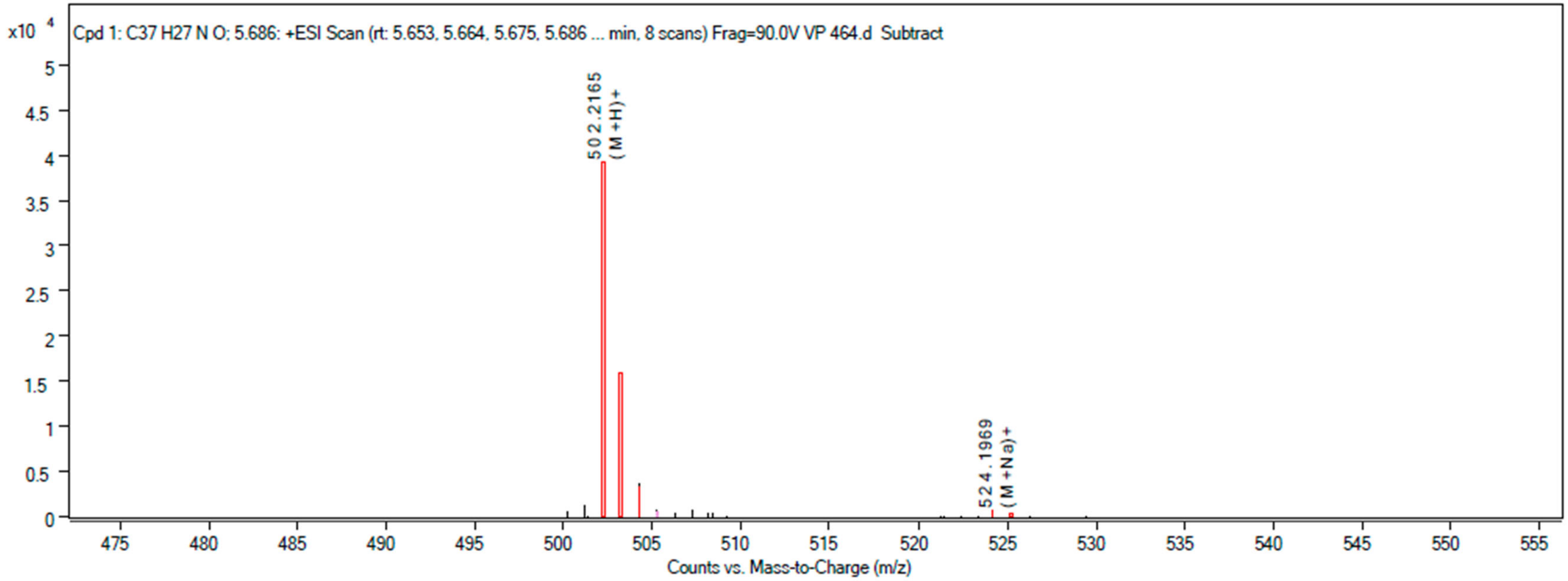

MS Spectrum Peak List

m/z	Calc *m/z*	Diff(ppm)	z	Abund	Ion
502.2165	502.2165	0.02	1	39346.6	(M+H)+
503.2192	503.2199	1.23	1	13096.46	(M+H)+
504.2257	504.2231	-5.17	1	3597.44	(M+H)+
524.1969	524.1985	2.99	1	705.27	(M+Na)+
525.199	525.2018	5.27	1	317.4	(M+Na)+
526.2114	526.2051	-12.01	1	29.74	(M+Na)+

--- End Of Report ---

Figure S11. HRMS (ESI) spectrum of compound **3**

Supporting info

Table S1. Summary of photophysical data for compounds **3, 4**

**Solvent/Media**	**3**		**4**	
	**λ_{Abs} (nm)**	**λ_{Em} (nm)**	**λ_{Abs} (nm)**	**λ_{Em} (nm)**
**DMF**	279, 329, 315, 346	380, 400, 421, 451	278, 328, 315, 345	381, 400, 421, 452
**DMF:H_2O [1:99 (*v/v*)]**	282, 320, 335, 353	389, 464	280, 318, 333, 350	384, 399, 470
**DMSO**	268, 279, 316, 330, 347	380, 400, 422, 463	268, 279, 316, 330, 347	381, 400, 423, 463
**DMSO:H_2O [1:1 (*v/v*)]**	267, 278, 315, 329, 345	381, 401, 424, 475	266, 278, 314, 329, 345	381, 400, 423, 473

Table S2. Excitation and emission wavelengths of compounds **3, 4** in fluorescence titration experiments in DMF and K_{SV} values and IFE correction calculations.

**Compound**	λ_{Ex}, nm	λ_{Em}, nm
**3**	346	451
**4**	345	452

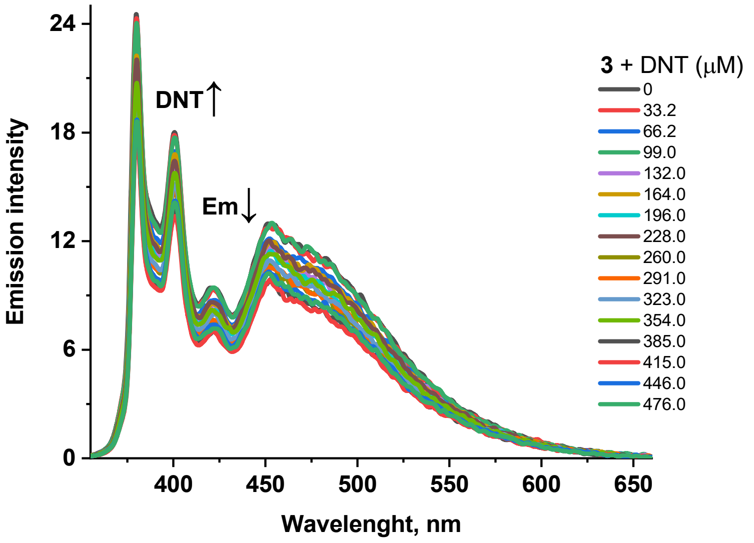

Figure S12. Overlayed spectra of fluorescence quenching of **3** upon addition of DNT in DMF.

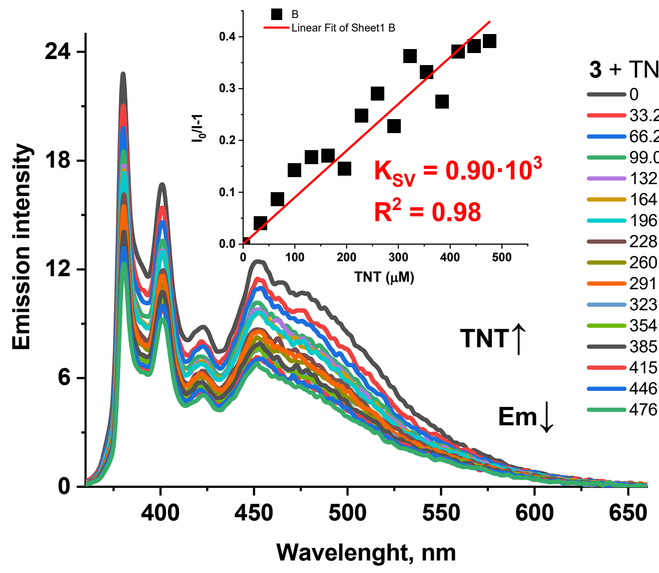

Figure S13. Overlayed spectra of fluorescence quenching of **3** upon addition of TNT in DMF; insert – Stern-Volmer plot of the fluorescence *"turn-off"* response of **3** to TNT after inner filter effect (IFE) correction.

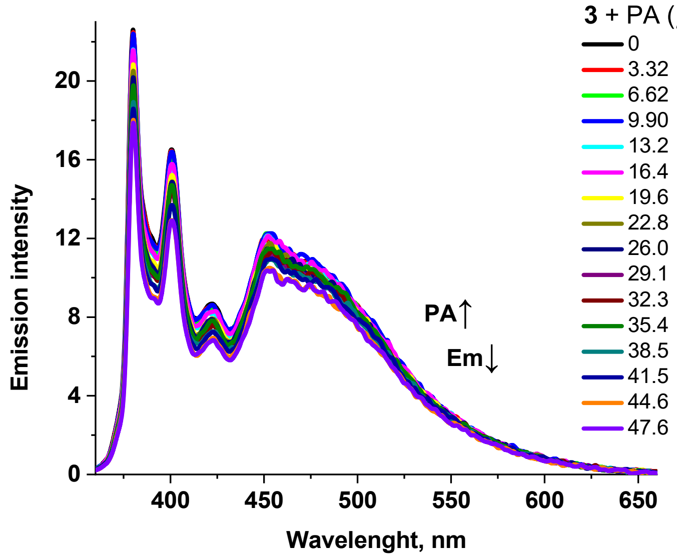

Figure S14. Overlayed spectra of fluorescence quenching of **3** upon addition of PA in DMF.

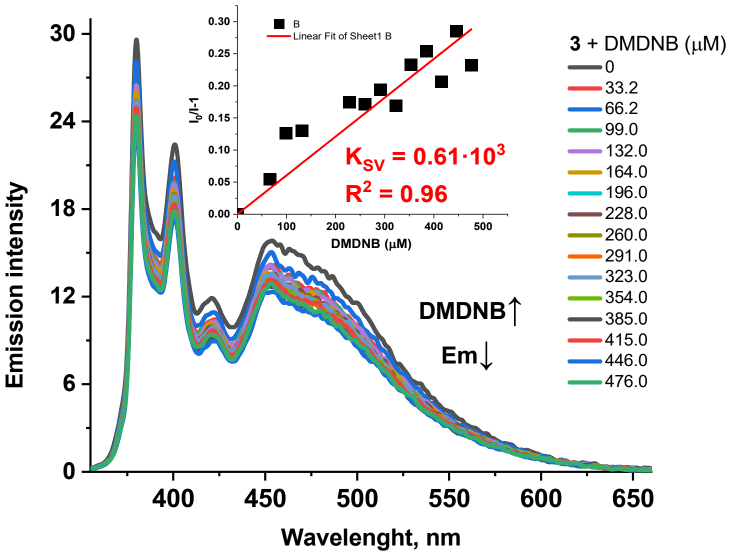

Figure S15. Overlayed spectra of fluorescence quenching of **3** upon addition of DMDNB in DMF; insert – Stern-Volmer plot of the fluorescence *"turn-off"* response of **3** to DMDNB after inner filter effect (IFE) correction.

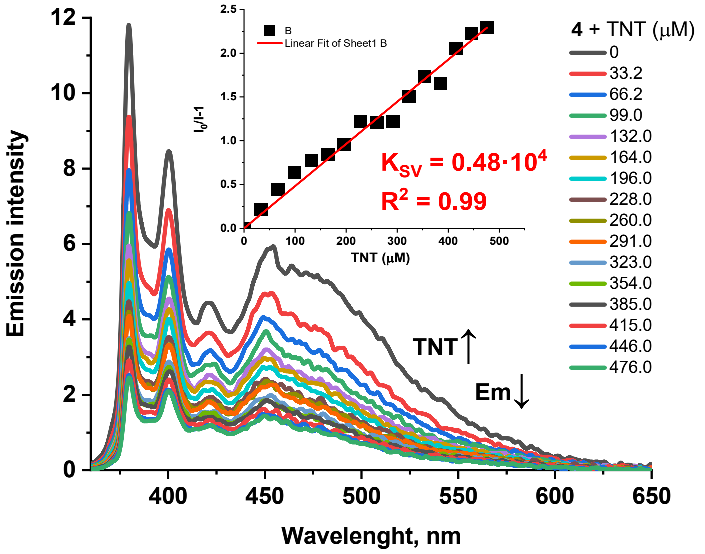

Figure S16. Overlayed spectra of fluorescence quenching of **4** upon addition of TNT in DMF; insert – Stern-Volmer plot of the fluorescence *"turn-off"* response of **4** to TNT after inner filter effect (IFE) correction.

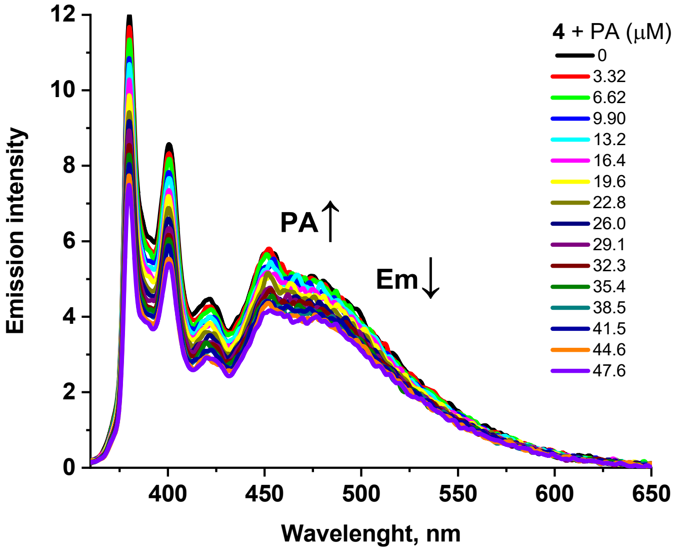

Figure S17. Overlayed spectra of fluorescence quenching of **4** upon addition of PA in DMF.

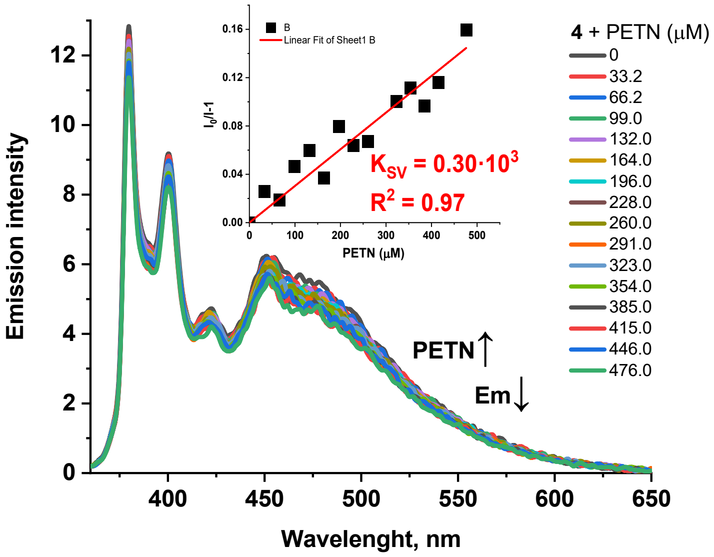

Figure S18. Overlayed spectra of fluorescence quenching of **4** upon addition of PETN in DMF; insert – Stern-Volmer plot of the fluorescence *"turn-off"* response of **4** to PETN after inner filter effect (IFE) correction.

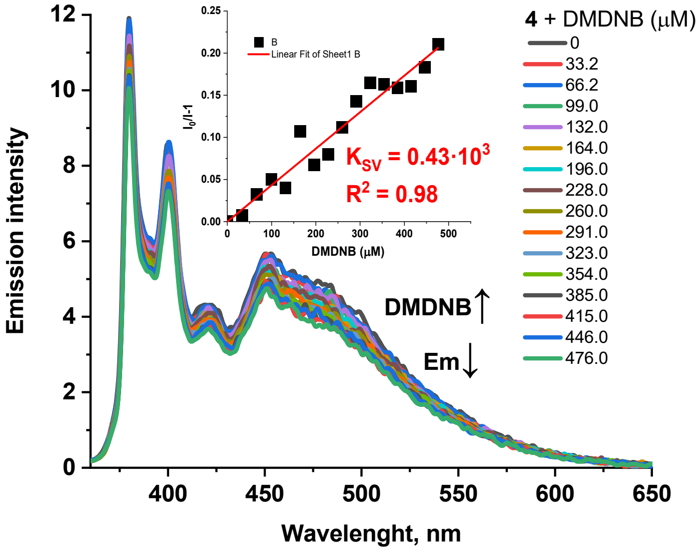

Figure S19. Overlayed spectra of fluorescence quenching of **4** upon addition of DMDNB in DMF; insert – Stern-Volmer plot of the fluorescence *"turn-off"* response of **4** to DMDNB after inner filter effect (IFE) correction.

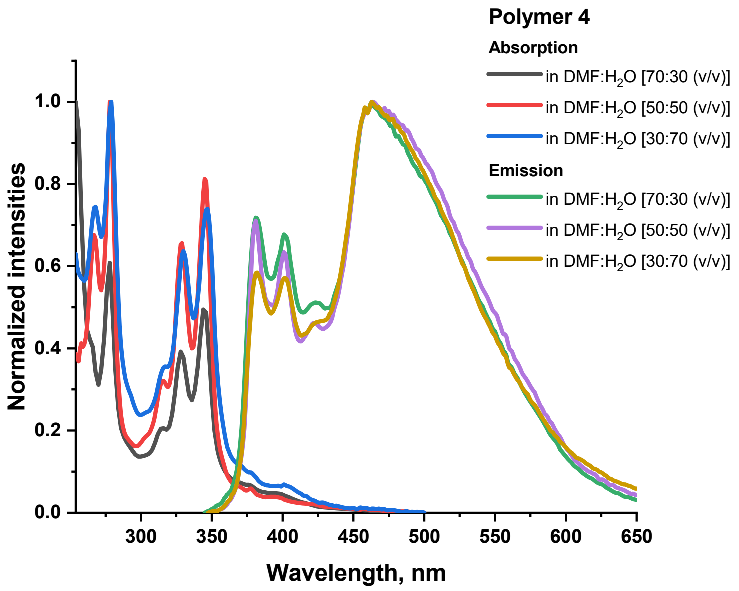

Figure S20. Normalized UV-Vis absorption and emission spectra of polymer **4** in DMF:H_2O [70:30 (*v/v*)], DMF:H2O [50:50 (*v/v*)] and DMF:H_2O [30:70 (*v/v*)].

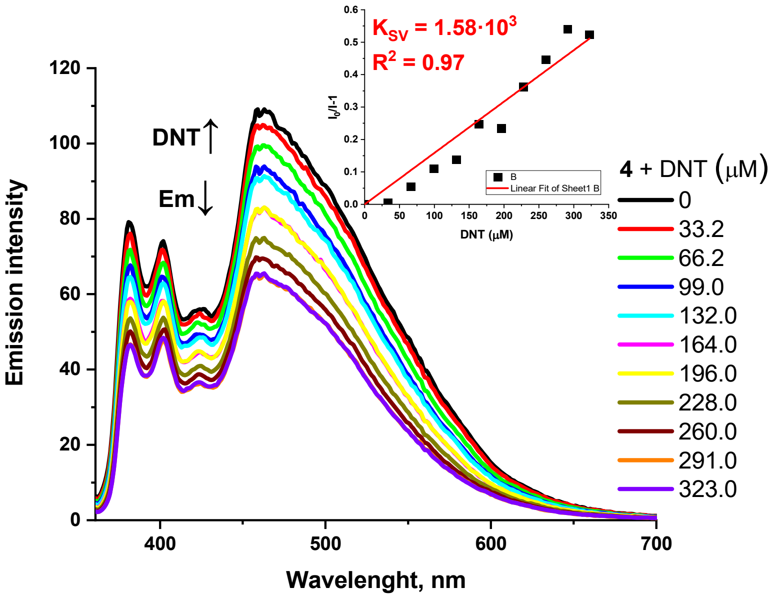

Figure S21. Overlayed spectra of fluorescence quenching of **4** upon addition of DNT in DMF:H_2O [70:30 (*v/v*)]; insert – Stern-Volmer plot of the fluorescence *"turn-off"* response of **4** to DNT after inner filter effect (IFE) correction.

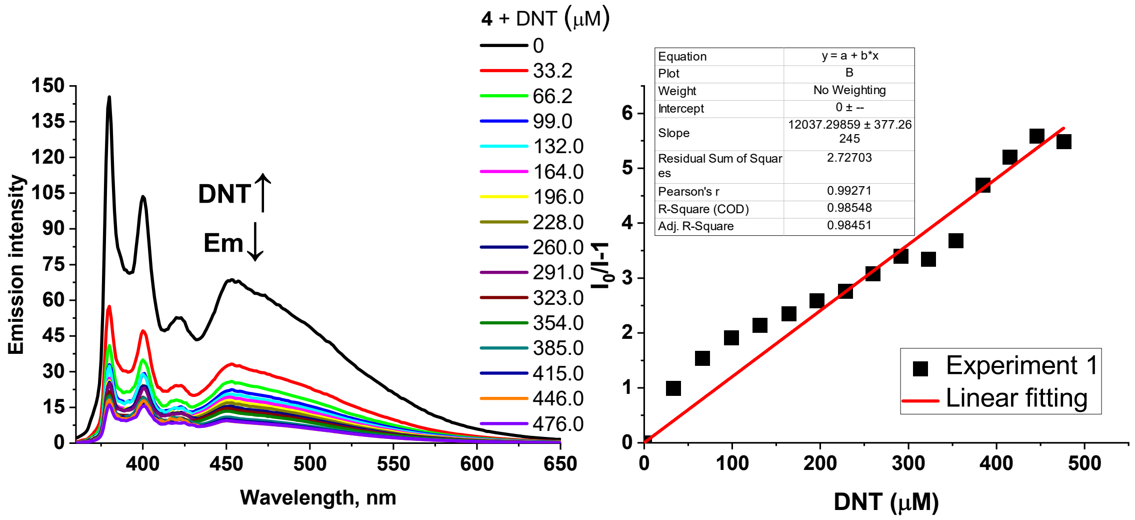

Figure S22. Overlayed spectra of fluorescence quenching of **4** upon addition of DNT in DMF (left); Stern-Volmer plot of the fluorescence *"turn-off"* response of **4** to DNT after inner filter effect (IFE) correction (right). The first parallel.

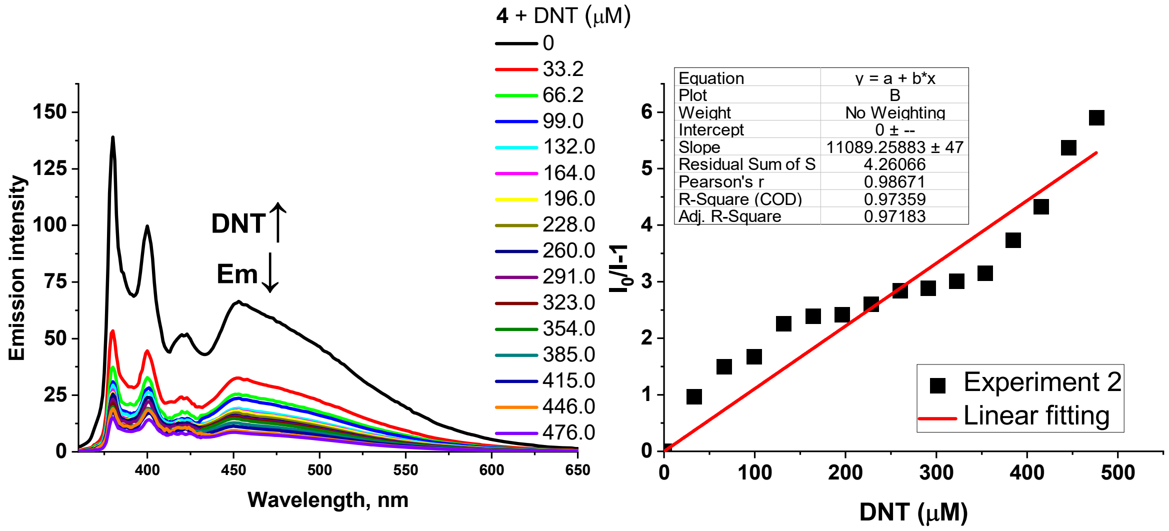

Figure S23. Overlayed spectra of fluorescence quenching of **4** upon addition of DNT in DMF (left); Stern-Volmer plot of the fluorescence *"turn-off"* response of **4** to DNT after inner filter effect (IFE) correction (right). The second parallel.

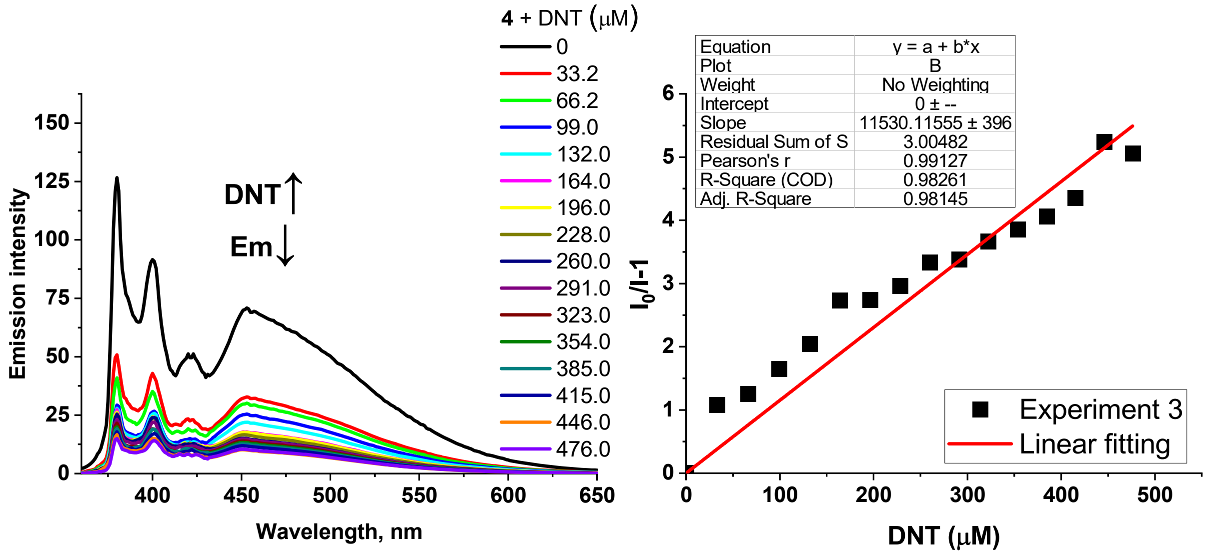

Figure S24. Overlayed spectra of fluorescence quenching of **4** upon addition of DNT in DMF (left); Stern-Volmer plot of the fluorescence *"turn-off"* response of **4** to DNT after inner filter effect (IFE) correction (right). The third parallel.

Sum, $\Sigma_x = 12037 + 11089 + 11530 = 34656$

Mean, $\mu = \frac{34656}{3} = 11552$

$$\sigma = \sqrt{\frac{1}{N}\sum_{i=1}^{N}(x_i - \mu)^2}$$

Supporting info

$$\sigma^2 = \frac{\sum(x_i - \mu)^2}{N} = \frac{(12037 - 11552)^2 + (11089 - 11552)^2 + (11530 - 11552)^2}{3}$$

$$= \frac{450078}{3} = 150026$$

$$\sigma = \sqrt{150026} = 387$$

K_{SV}, calculated from three experiments = 11552 ± 387 M^{-1}
